# Multiphoton Quantum Reservoirs For Robust Multidimensional Computing

**DOI:** 10.1002/advs.77225

**Published:** 2026-08-19

**Authors:** Mingyuan Hong, Mario A. Quiroz‐Juarez, Riley B. Dawkins, Fatemeh Mostafavi, Jannatul Ferdous, Addison Wilberg, Blas M. Rodriguez‐Lara, Justin Woodring, Chenglong You, Roberto de J. León‐Montiel, Omar S. Magaña‐Loaiza

**Affiliations:** ^1^ Quantum Photonics Laboratory, Department of Physics & Astronomy Louisiana State University Baton Rouge Louisiana USA; ^2^ Centro de Física Aplicada y Tecnología Avanzadas Universidad Nacional Autónoma de México Querétaro Mexico; ^3^ Universidad Politécnica de Pachuca Zempoala Hidalgo Mexico; ^4^ School of Electrical Engineering and Computer Science Louisiana State University Baton Rouge Louisiana USA; ^5^ Instituto de Ciencias Nucleares Universidad Nacional Autónoma de México Mexico City México

**Keywords:** photonic lattices, photonic network, quantum simulator, reservoir computing

## Abstract

One of the most significant achievements of the second quantum revolution is enabling tasks that are infeasible for classical computers. However, the stringent requirements for quantum resources, along with the presence of noise and losses, impose limitations on technologies for quantum information processing. Here, we overcome these limitations by demonstrating robust and scalable quantum reservoirs for multiphoton quantum computing, the first of their kind to operate robustly at room temperature under high levels of noise and decoherence. By implementing Fock projective measurements, we extract multiphoton quantum systems from classical fields, enabling the engineering of quantum reservoirs that are robust to losses and noise. Our multiphoton reservoirs support universal quantum information processing with up to forty particles, exhibiting quantum speedups over classical counterparts. Remarkably, the multiparticle interactions hosted by our reservoirs enable us to perform quantum simulations of nonlinear systems. Specifically, we simulate the quantum thermodynamics of many‐body systems in synthetic lattices. Furthermore, we exploit the complexity of our multiphoton quantum reservoir, consisting of eight hundred sixty‐one components, for the quantum prediction of mathematical functions. As such, our work unveils a path toward the development of robust quantum technologies for information processing, long regarded as a central goal of the field.

## Graphical abstract

Projective measurements reveal hidden multidimensional quantum networks within classical multiphoton fields, enabling robust quantum reservoirs for room‐temperature quantum simulation, prediction, and information processing. These networks transform noisy classical photonic platforms into scalable quantum computational architectures.

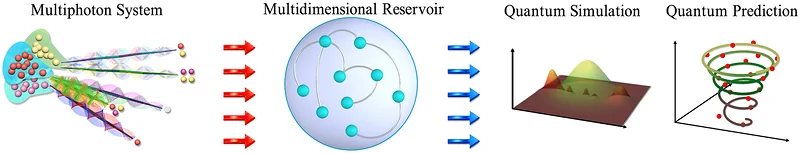

The realization of robust quantum technologies hinges on the efficiency of linear networks in implementing unitary transformations on nonclassical systems [1, 2, 3]. The implications of demonstrating robust quantum networks have motivated extensive exploration of various platforms, including trapped atoms, quantum dots, superconducting qubits, nitrogen‐vacancy centers, and photonic systems [4, 5, 6, 7]. Notably, the possibility of observing quantum properties of photons at room temperature makes them particularly advantageous for the implementation of robust quantum networks [1, 4, 8, 9]. In particular, quantum photonic networks have the potential to host large multiparticle systems with numerous interactions, which enable efficient quantum interference, teleportation, and entanglement swapping [2, 3, 10]. Such capabilities are crucial for demonstrating realistic quantum information processing and simulation [10, 11, 12]. Despite the potential of quantum photonic networks for quantum information, their scalability remains fundamentally limited [13]. To some extent, this constraint arises from quantum multiphoton sources that generate only a few particles [4, 13, 14, 15]. Furthermore, the fragility of these platforms against noise and losses imposes additional challenges, preventing the practical realization of quantum photonic computers [1, 4, 9, 10, 16].

The limitations of existing quantum networks have led to the exploration of new ways to process quantum information, one of which uses a concept called reservoir computing [17, 18, 19]. This alternative computing scheme goes beyond von Neumann architectures [20]. In the context of quantum information, this has inspired the development of quantum reservoir computing [20, 21, 22, 23, 24, 25, 26, 27]. This paradigm employs stochastic, highly‐interconnected quantum networks as reservoirs, which enable the storage and processing of information without direct control over the hosting physical system [20]. This feature of quantum reservoir computing makes it more scalable and efficient than conventional schemes for quantum information processing [20, 21, 22, 24, 25, 26, 27]. Nevertheless, the demonstration of a photonic platform for quantum reservoir computing remains elusive. Remarkably, recent findings suggest the potential of using the nonlinearity of projective measurements in the Fock basis to isolate multiparticle systems [28, 29]. This measurement scheme enables the extraction of multiphoton quantum states from classical light sources [28, 29, 30, 31]. While these effects have been explored in the context of nanophotonic systems and quantum sensors [28, 29, 30], their implications for multiphoton quantum information processing have yet to be investigated.

Here, we report the first demonstration of robust multiphoton quantum reservoirs that take advantage of the inherent loss insensitivity of thermal systems [16, 34]. This is accomplished by extracting bosonic or fermionic quantum multiphoton subsystems from classical thermal wavepackets, which generate distinct quantum reservoirs through Fock projective measurements using photon‐number‐resolving (PNR) detection [14, 35, 36]. These projective measurements provide nonlinearities that are essential for quantum reservoir computing [17, 18, 20]. Remarkably, isolating the vacuum dynamics of our reservoir enables us to convert classical information‐processing routines, affected by decoherence, into their quantum counterparts [29]. We demonstrate that this possibility allows one to speed up quantum information processing using bosonic or fermionic multiphoton systems containing up to forty particles. The multiphoton coupling within our multidimensional reservoirs enables quantum simulations of nonlinear systems, allowing us to simulate the quantum thermodynamics of many‐body systems in synthetic lattices [32, 33, 37]. Finally, we exploit the complexity of our multiphoton quantum reservoir, consisting of eight hundred sixty‐one components, to implement quantum predictions of nontrivial mathematical functions. Our results demonstrate the robustness of hybrid quantum technologies against decoherence, particularly those that combine classical light sources with nonclassical multiphoton detection. As such, we believe our work has significant implications for the implementation of quantum protocols in realistic environments where losses and noise are unavoidable [16].

Our platform relies on multiphoton systems with different orbital angular momentum (OAM) and polarization properties [38, 39]. As illustrated in Figure 1a, the various degrees of freedom of our platform enable us to produce multidimensional quantum reservoirs, which we access through projective measurements [28, 30, 31]. We exploit the coupling supported by this type of network to perform simulations of quantum thermodynamics and to enable quantum predictions of mathematical functions. Specifically, the unitary operator of our quantum network is given by the multi‐layer transformation

(1) $$\hat{U}=\sum_{n,m}\gamma_{n,m}\left(\vec{\eta }\right)|n\rangle \langle m|,$$

where the n,m are vectors representing the number of quanta in each polarization‐OAM mode. In addition, $\vec{\eta }$ is a (1,2)‐tensor representing the configuration of each layer of the reservoir, and $\gamma_{n,m}\left(\vec{\eta }\right)$ are the matrix coefficients of the reservoir. As shown in Note S1, these coefficients are given explicitly by

(2) $$\begin{array}{cc} \gamma_{n,m}\left(\vec{\eta }\right)= & \sum_{\begin{array}{c} I_{n}\\ \forall \lambda^{\prime \prime },\lambda^{\prime \prime }\left[I_{n}\right]=m_{\lambda^{\prime \prime }} \end{array}}\left(\prod_{\lambda }\sqrt{\frac{m_{\lambda }!}{n_{\lambda }!}}\right)\\ & \times \;\left(\prod_{I_{n_{\lambda }}\in I_{n}}\prod_{\lambda^{\prime }\in I_{n_{\lambda }}}u_{\lambda \lambda^{\prime }}\right). \end{array}$$

Here, we use three types of multi‐indices. The first kind corresponds to a single mode, denoted by λ≡(s,ℓ,p) for polarization s and OAM configuration (ℓ,p). The second is the multi‐index $I_{n_{\lambda }}$, representing a sequence of $n_{\lambda }$ modes $\lambda^{\prime }$, where the sum over this multi‐index is over all such sequences. Furthermore, we have the multi‐index $I_{n}\equiv \left(I_{n_{\lambda_{1}}},I_{n_{\lambda_{2}}},\text{…}\right)$ of all such multi‐indices $I_{n_{\lambda }}$, which correspond to each $n_{\lambda }$ in the vector n. We have also introduced the combinatorial function $\lambda^{\prime \prime }\left[I_{n}\right]$, which counts the total number of times $\lambda^{\prime \prime }$ occurs throughout all of the $I_{n_{\lambda }}\in I_{n}$. Finally, the $u_{\lambda \lambda^{\prime }}$ terms in Equation (2) are obtained by evolving each mode's creation operator through the quantum reservoir, a process which is denoted by ${\hat{a}}_{\lambda }^{†}\to \sum_{\lambda^{\prime }}u_{\lambda \lambda^{\prime }}{\hat{a}}_{\lambda^{\prime }}^{†}$. These coefficients can be calculated directly from $\vec{\eta }$. We note that computing the output of our quantum reservoir involves the calculation of the permanent of submatrix of $\hat{U}$ shown in Equation (1) [40]. Our system is equivalent to the Gaussian boson sampling problem, which is well‐known to be #P‐complete [9]. Consequently, the high‐dimensional multiphotonic network described by Equation (2) constitutes our quantum reservoir, formed by the coupled polarization, OAM, and photon‐number degrees of freedom, together with the nonlinear readout induced by photon‐number projective measurements. The complexity of this transformation, combined with the nonlinearity introduced by photon‐number projection, enables this quantum network to emulate simpler quantum systems, including synthetic lattices, random‐walk networks, and quantum reservoir computing architectures. We demonstrate these capabilities below. The application of this theoretical framework to our experimental wave‐plate and q‐plate network, including the derivation of the photon‐number‐resolved output probabilities obtained through Fock‐state projection, is presented in Note S2. The effective Hamiltonian governing the thermalization and anti‐thermalization dynamics discussed below is derived in Note S3.

**FIGURE 1:**
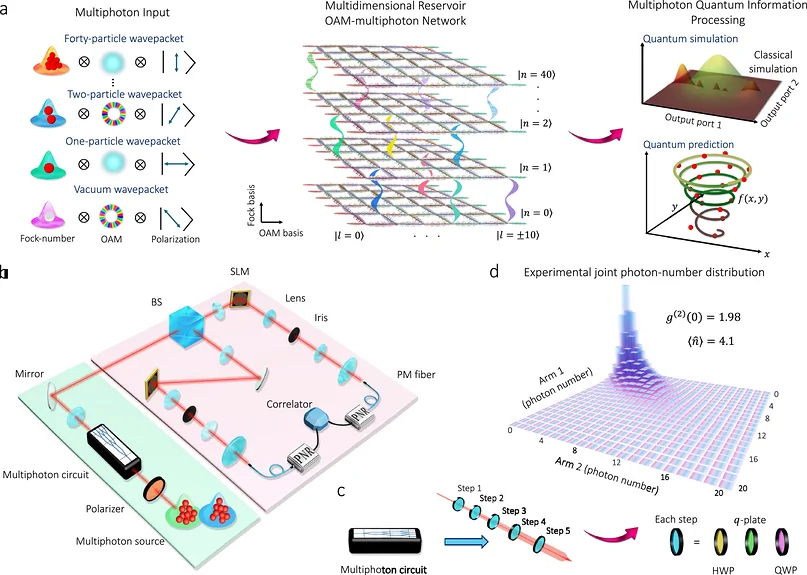
Multiphoton quantum reservoirs for multidimensional information processing. As shown in (a), our multiphoton reservoir‐based platform for quantum information processing exploits the Fock particle number, orbital angular momentum (OAM), and polarization of classical light fields. The distillation of quantum multiphoton systems enables the engineering of multidimensional networks, which we use to implement quantum simulations and prediction of nontrivial mathematical functions. This robust platform is explored using the experimental setup depicted in (b). Here, we inject a classical multiphoton field with a specific polarization into our photonic circuit. The emerging field from the circuit is then passed through a beam splitter (BS) and subsequently characterized by performing projective measurements in both the spatial OAM and photon‐number bases. The OAM of the multiphoton wavepackets is measured using spatial light modulators (SLMs), while the photon‐number distributions are reconstructed using photon‐number‐resolving (PNR) measurements implemented with avalanche photodiodes (APDs) connected to a time tagger. Further details of the APD time‐tagging implementation are provided in Note S4, while the fidelity and scalability of the time‐binned photon‐number‐resolving measurement are quantified in Note S5. As illustrated in (c), our photonic circuit consists of five steps. Each step consists of a half‐wave plate (HWP), a q‐plate, and a quarter‐wave plate (QWP). Our q‐plates increase or decrease the OAM of the injected multiphoton wavepackets by one or two units, depending on their topological charge q. Furthermore, each q‐plate modifies the polarization components of the multiphoton wavepacket, converting left‐handed polarization to right‐handed, and vice versa. The experimental joint photon‐number distribution of the injected forty‐particle multiphoton system is presented in (d). The degree of second‐order coherence $g^{\left(2\right)}\left(0\right)=1.98$ indicates that the input field exhibits classical statistics, with its brightness characterized by a mean photon number of 4.10. The robustness of our platform resides in our ability to isolate multiphoton quantum systems from classical fields, whose properties remain unaffected by losses in the multiphoton network.

Our experimental demonstration of multiphoton reservoir computing is performed using the setup depicted in Figure 1b. We inject a classical multiphoton beam into a free‐space tunable photonic circuit. As shown in Figure 1c, each step or layer in our circuit is implemented by combining a half‐wave plate (HWP) with a q‐plate and a quarter‐wave plate (QWP) [39]. Each step increases or decreases the OAM of the injected multiphoton system through the spin–orbit coupling introduced by the q‐plate. The effective coupling among the polarization, OAM, and photon‐number degrees of freedom emerges from the complete linear optical network and does not rely on any material‐mediated optical nonlinearity (see Notes S1 and S4). In this setup, each node in the circuit corresponds to a specific OAM value with distinct polarization properties. The field emerging from the circuit is split into two replicas, each of which is then projected onto OAM modes using spatial light modulators (SLMs) [41]. The light fields reflected from the SLMs are subsequently measured by two photon number‐resolving (PNR) detectors, enabling projective measurements on different Fock states [28, 29]. In our implementation, PNR measurements are implemented using the time‐tagged outputs of Excelitas SPCM‐AQRH‐14‐FC avalanche photodiodes using a PicoQuant MultiHarp 150 time tagger. The detection records are grouped into 1 μs measurement windows, and the photon number associated with a given window is determined from the registered detection events within that window. The experimental implementation is described in Note S4, and a quantitative analysis of the fidelity and scalability of this time‐binned photon‐number‐resolving scheme is provided in Note S5, with the corresponding detector‐fidelity analysis shown in Figure S2. Importantly, photon‐number projective measurements allow us to resolve and selectively extract the constituent multiparticle systems associated with different photon‐number outcomes of the same host field. Thus, multiphoton events are not restricted to one‐ or two‐photon outcomes; rather, PNR detection provides access to a family of multiparticle subsystems through conditional Fock‐state projection. This photon‐distillation procedure underlies the multiparticle wavepackets used throughout this work (see Note S6). Using this measurement technique, we extract the quantum multiphoton systems constituting the injected classical field shown in Figure 1d. Here, we present a thermal forty‐particle system characterized by a degree of second‐order coherence $g^{\left(2\right)}\left(0\right)$ of 1.98 and a mean photon number $\left\langle \hat{n}\right\rangle$ of 4.1.

We first use our multiphoton reservoir to implement a quantum random walk (QRW) with a single PNR detector [42]. Our interest in this configuration lies in its relevance as a universal computational primitive for quantum simulation and quantum search algorithms [1, 10, 42]. Interestingly, the presence of coherence in QRWs provides a quadratic computational advantage or speedup over classical random walks (CRWs) [8, 43]. This advantage is quantified through the standard deviation of the photons emerging from the reservoir. However, these circuits are fragile in the presence of noise, which can induce decoherence in the photonic network, ultimately reducing a QRW to a CRW [8, 9]. We alleviate this limitation by extracting the constituent quantum multiparticle systems from the classical thermal light field injected into the photonic network. To achieve this, we use the five‐step quantum network, depicted in Figure 2a, within our reservoir to implement a QRW protocol under noisy conditions. For this task, the reservoir is operated in an ordered configuration. The HWP placed between successive OAM‐shifting operations reverses the circular‐polarization flip introduced by each q‐plate, causing the two branches of the walk to continue along opposite OAM directions. Consequently, after the xth nominal step, the two ballistic branches occupy the OAM components ℓ=+x and ℓ=−x (see Note S4). Despite the noise, the presence of coherence within the reservoir gives rise to an imperfect ballistic spreading, as shown in Figure 2b. Interestingly, our ability to perform projective measurements in the Fock number basis enables us to attenuate the noise levels in the network to obtain a nearly perfect ballistic distribution. The purpose of this measurement is not to show that ballistic spreading requires multiple photons. Ballistic transport can indeed be observed in the single‐photon regime. Here, the measurement demonstrates that photon‐number projection can isolate higher‐order multiphoton components from a noisy thermal field and recover their corresponding QRW distributions. This is demonstrated by projecting the emerging thermal light field from the network into a seven‐particle subsystem. Experimentally, the seven‐particle QRW is obtained by retaining the n=7 outcomes for each measured OAM projection and normalizing the resulting counts over the measured OAM modes, yielding the conditional distribution P(ℓ|n=7). It therefore represents the OAM distribution conditioned on the seven‐particle component of the output field, rather than a seven‐fold coincidence measurement from seven independently prepared single photons (see Note S4). This operation can be described in terms of the unitary transformation in Equation (1) as ${\hat{\rho }}_{\text{out}}={\hat{U}}^{†}{\hat{\rho }}_{\text{in}}\hat{U}$. From here, a measurement of the output state $|n\rangle$ will have a probability given by $\sum_{m_{1},m_{2}}\gamma_{m_{1},n}^{*}\gamma_{m_{2},n}\langle m_{1}|{\hat{\rho }}_{\text{in}}|m_{2}\rangle$ (see Notes S1 and S4). The superior quantum performance of our photonic network, compared to its classical counterpart, is reported in Figure 2c. As expected, the classical or incoherent operation of our reservoir induces diffusive spreading, which results in the Gaussian distributions seen in Figure 2d [1, 43]. Remarkably, projecting these Gaussian distributions from the CRW into a vacuum state and other Fock states enables us to navigate the number basis of our multidimensional reservoir shown in Figure 1a to extract the quantum constituents of an otherwise classical walk. Furthermore, this measurement technique allows for the isolation of bosonic and fermionic multiphoton systems [44], thereby enabling the simulation of many‐body dynamics governed by bosonic and fermionic exchange statistics on a single platform [28, 29]. This represents an ambitious goal in the advancement of quantum technologies, one that has thus far remained elusive [45, 46]. As demonstrated in Figure 2e, here we simulate bosonic and fermionic systems in our multiphoton reservoir. In Figure 2e, $n_{1}$ and $n_{2}$ denote the photon numbers conditionally selected in detection arms 1 and 2, respectively. The plotted distributions are normalized joint OAM distributions conditioned on the selected photon‐number pair $\left(n_{1},n_{2}\right)$. Specifically, we implemented a QRW using two PNR detectors to produce ballistic bosonic spreading with twenty‐six ($n_{1}=13$, $n_{2}=13$) and forty‐two ($n_{1}=21$, $n_{2}=21$) photons. We also demonstrate the first multiparticle simulation of anti‐correlated QRWs produced by fermionic statistics. This is experimentally demonstrated for photonic systems with eight ($n_{1}=7$, $n_{2}=1$) and fourteen ($n_{1}=13$, $n_{2}=1$) photons. These effects demonstrate the potential of our multidimensional network to overcome decoherence in platforms for quantum information processing.

**FIGURE 2:**
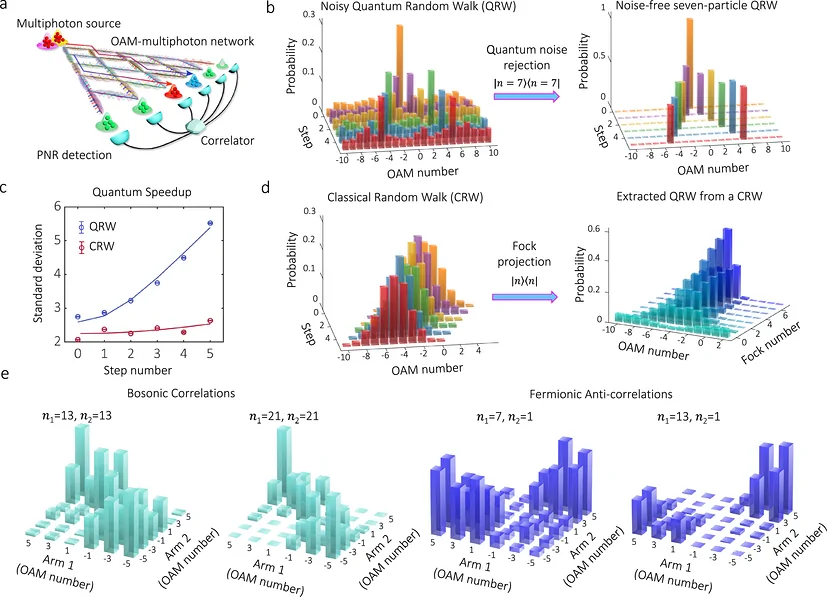
Extracting quantum information from classical multiphoton reservoirs in the presence of noise. The robustness of our multiphoton reservoir to noise and losses is explored through the implementation of the five‐step classical and quantum random walk using the network illustrated in (a). We first implement the quantum random walk (QRW) protocol in a noisy environment. The presence of coherence in the multidimensional reservoir leads to the imperfect ballistic spreading reported in the first panel of (b). Interestingly, the projection into a seven‐particle system results in a nearly perfect ballistic distribution. The superior performance of the QRW, compared to its classical counterpart, is quantified by the standard deviation of the distribution at the output of the reservoir, as shown in (c). Furthermore, the classical or incoherent operation of our reservoir induces diffusive spreading, resulting in the Gaussian photon distributions shown in the first panel of (d). These distributions are associated with classical random walks (CRWs). Remarkably, the multidimensional nature of our reservoir enables us to navigate through the number basis by performing projections into Fock states to extract the quantum constituents of CRWs. We demonstrate this possibility by projecting the Gaussian distribution obtained in the fourth step of the network. This effect illustrates the potential of our multidimensional network to overcome decoherence in platforms for quantum information processing. As reported in (e), the implementation of a QRW with two PNR detectors enables us to produce ballistic bosonic spreading with twenty‐six ($n_{1}=13$, $n_{2}=13$) and forty‐two ($n_{1}=21$, $n_{2}=21$) photons. Notably, we also demonstrate the simulation of an anti‐correlated QRW produced by fermionic statistics. This is experimentally demonstrated for photonic systems with eight ($n_{1}=7$, $n_{2}=1$) and fourteen ($n_{1}=13$, $n_{2}=1$) photons.

To demonstrate the capability of our multidimensional reservoirs for quantum simulation, we explore the thermalization and anti‐thermalization of many‐body systems in photonic lattices [32, 33, 47, 48]. While this phenomenon has been extensively studied in the context of quantum thermodynamics, it has thus far only been emulated using classical multimode photonic systems and nonlinear interactions [32, 33]. Here, we use our multiphoton reservoir to simulate thermalization and anti‐thermalization dynamics of wavepackets [32, 33, 47]. These interesting dynamics are described by the Hamiltonian operator

(3) $$\begin{array}{cc} \hat{H}= & \left(\omega_{0}+i\frac{{\dot{A}}^{*}}{A^{*}}\right){\hat{a}}^{†}\hat{a}+i\left(\dot{B}-B\frac{{\dot{A}}^{*}}{A^{*}}+i\omega_{0}B\right){\hat{a}}^{†}\\ & -i\left({\dot{B}}^{*}-B^{*}\frac{\dot{A}}{A}+i\omega_{0}B^{*}\right)\hat{a}, \end{array}$$

here, the function A(t) describes a changing intensity, and the function B(t) models a coherent displacement process (see Note S3). The annihilation operator $\hat{a}$ represents the observable Fock space, which is obtained by projecting into a particular OAM value, and $\omega_{0}$ is the frequency of the multiphoton wavepacket. The effect of this evolution on our quantum state is best‐described in terms of the Glauber–Sudarshan P‐function representation [49, 50], and is given by

(4) $$\begin{array}{cc} {\hat{\rho }}_{\bar{n},\mu }\left(t\right) & =\int d^{2}\alpha \frac{1}{{\pi |A\left(t\right)|}^{2}\bar{n}}\\ & \times \exp\left[-\frac{|\alpha -\mu A\left(t\right)e^{i\omega_{0}t}{-B\left(t\right)|}^{2}}{\bar{n}{\left|A\left(t\right)\right|}^{2}}\right]|\alpha \rangle \langle \alpha |. \end{array}$$

We use $\bar{n}$ to describe the initial thermal amplitude of the wavepacket and μ to account for its initial coherent amplitude. Our multiphoton reservoir enables us to control A(t) and B(t) to perform quantum simulation of thermalization or anti‐thermalization (detailed derivation of this effect can be found in Note S3). In our experiment, we utilize a quantum reservoir to implement the Hamiltonian in Equation (3) to manipulate the excitation mode of a multiphoton wavepacket containing up to twenty particles. This capability marks the first demonstration of quantum simulation of many‐body thermalization dynamics. To this end, we designed the synthetic lattice shown in Figure 3a, where each node corresponds to a specific OAM number capable of hosting multiparticle wavepackets. We demonstrate that propagation along particular paths in the lattice gives rise to distinct thermalization dynamics in multiparticle systems [51]. The network configuration used for this experiment differs from the ordered QRW configuration. Between successive q‐plates, the combined HWP and QWP transformations generally prepare superpositions of left‐ and right‐circular polarization. The subsequent spin–orbit coupling therefore distributes the field over a richer set of positive and negative OAM pathways, producing the branching synthetic lattice shown in Figure 3a. Photon‐number statistics are then measured at selected lattice nodes, and the corresponding marginal distributions are used to evaluate $g^{\left(2\right)}\left(0\right)$ along the thermalization and anti‐thermalization trajectories (see Note S4). As shown in Figure 3b, propagation along the path i–ii–iii attenuates the quantum fluctuations of the wavepacket, thereby reducing its noise properties. The modification in the quantum statistics of the wavepacket is described by Equation (4). Remarkably, this process occurs upon propagation in the absence of optical nonlinearities and, more generally, without any light‐matter interactions. The suppression of chaotic fluctuations upon propagation through the synthetic lattice enables the exploration of anti‐thermalization dynamics in our platform [52]. Interestingly, the same lattice enables us to simulate the thermalization of the wavepacket, leading to an amplification of the chaotic fluctuations in the multiparticle field. This effect is confirmed by measuring an increasing degree of second‐order coherence $g^{\left(2\right)}\left(0\right)$, in each detector, through the propagation of the I‐II‐III path. The evolution of the wavepacket is reported in Figure 3c. The ability of our quantum reservoir to support these quantum dynamics has significant implications for quantum thermodynamics. Thus far, these effects have only been investigated through classical optical analogs [32, 33].

**FIGURE 3:**
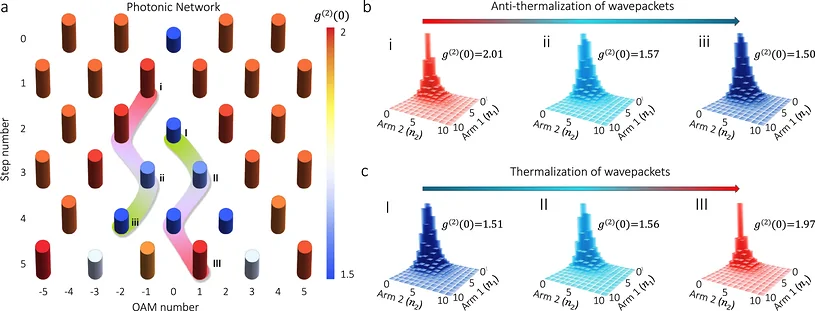
Multidimensional quantum simulation of thermalization dynamics in photonic lattices. Utilizing our multidimensional reservoir, we have implemented the synthetic lattice depicted in (a). Each node in this lattice represents a specific OAM number and can accommodate multiphoton wavepackets containing up to twenty particles. Remarkably, this multidimensional architecture enables us to explore the anti‐thermalization and thermalization of many‐body systems in the absence of optical nonlinearities [32, 33]. Specifically, we demonstrate the attenuation of quantum fluctuations in a wavepacket propagating along the i‐ii‐iii path. The joint photon‐number distributions in (b) illustrate how our reservoir enables the simulation of wavepacket propagation in a lattice. As described by Equation (4), this capability allows for the exploration of modifications to the excitation mode of a multiparticle system to suppress noise. This is certified by a decreasing degree of second‐order coherence $g^{\left(2\right)}\left(0\right)$. Note that second‐order coherence is computed by means of the marginal photon distribution in each detecting arm. Notably, propagation through different nodes in the lattice enables us to modify the Fock‐state distribution of the wavepacket. The panels in (c) show that the wavepacket propagating along the I‐II‐III path exhibits increased quantum fluctuations. Given the nonclassicality of number states, the thermalization process that we simulate using our network is entirely quantum.

Finally, we exploit the high connectivity and complexity of our reservoir to demonstrate robust multiphoton computing [19]. We achieve this through the implementation of computational protocols rooted in recurrent neural network theory [17, 18, 20]. We demonstrate the potential of our platform to perform complex tasks by using random multiphoton reservoirs to map input signals into higher‐dimensional spaces, which are then optimized by low‐level neural networks [18]. The projective measurements in the Fock basis provide nonlinearities that are essential for quantum reservoir computing [53]. This approach enables the reconstruction of arbitrary functions, $F(\bar{x})$, using a known input vector $\bar{x}$. We outline our photonic platform for reservoir computing in Figure 4a. The protocol entails encoding the input vector $\bar{x}$ in the linear polarization states of a single‐mode spatially coherent light beam. We use ninety‐one distinct linear polarization states, ranging from horizontal to vertical polarization. The elements of the input vector are then injected into our quantum reservoir, which is prepared by randomly selecting the orientations of the HWPs and QWPs in each step of the multiphoton circuit (see Notes S1, S2 and S4). Once selected, the internal HWP and QWP orientations are held fixed throughout the complete measurement so that all ninety‐one polarization‐encoded inputs are processed by the same physical reservoir. Complex coupling between the OAM and multiphoton components of the coherent state—mathematically described by the transformations in Equations (1) and (2)—takes place within this setup. We create a higher‐dimensional computational state by monitoring the probability of detecting up to forty photons in each of the twenty‐one OAM modes supported by our photonic reservoir. As such, our quantum reservoir effectively generates an output feature vector with 861 components for each polarization‐encoded input value. For each input polarization, we measure twenty‐one OAM projections, ℓ=−10,…,10. Each polarization‐OAM setting is acquired for 1 s and analyzed using $10^{6}$ measurement windows of 1 μs. The full task‐specific configuration and data‐acquisition procedure are described in Note S4. Representative photon‐number distributions used to construct the reservoir output are shown in Figure S1. We use this multidimensional output to train (80% of the data) and validate (20%) a regression network, which employs a single layer of linear‐activation neurons to learn the target function $F(\bar{x})$ specified by the user. We present the results of our multiphoton quantum reservoir computing in Figure 4b through 4g, which correspond to six different target functions: quadratic, cubic, quartic, exponential, logarithmic, and damped sinusoidal. The training data are represented by blue diamonds, while the quantum predictions are shown as red dots. The gray solid line represents the target function. Notably, all six functions shown in Figure 4b–g are computed using the same multiphoton quantum reservoir. This highlights that, despite being static, our physical quantum reservoir is sufficiently complex to simultaneously interpolate, with high accuracy, functions with distinctly different behaviors.

**FIGURE 4:**
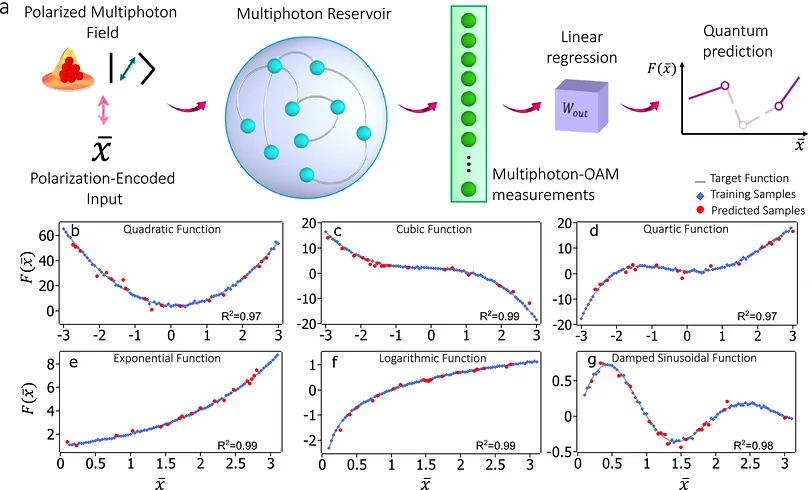
Multiphoton quantum reservoir computing. The implementation of our multiphoton protocol for quantum reservoir computing is depicted in (a). Here we encode the input vector $\bar{x}$ in the linear polarization states of a single‐mode spatially coherent light beam, spanning ninety‐one distinct polarization angles from horizontal to vertical. The elements of the input vector are fed into our quantum reservoir where the coupling between OAM and photon‐number components of the photonic system takes place. A higher‐dimensional output feature vector is then created by monitoring the probability of detecting up to forty photons in each of the twenty‐one OAM modes supported by the photonic network. The quantum reservoir effectively maps each polarization‐encoded input into an output feature vector with eight hundred sixty‐one components. We allocate 80% of the data for training and the remaining 20% for validation, using a linear regression network tasked with learning the target function $F(\bar{x})$. In the panels from (b–g), we show the results for quantum reservoir computing of six different target functions: quadratic, cubic, quartic, exponential, logarithmic, and damped sinusoidal, respectively. The training data is depicted by blue diamonds, while the quantum predictions are represented by red dots. The gray solid line depicts the target function, providing a visual comparison of the learned mapping.

To test the quantum enhancement that our platform provides, we characterize its computational performance and compare it with that of its classical counterpart in Note S7 [18, 19]. In this comparison, the classical reservoir is built from the same optical field but retains only the mean photon number of each OAM mode, discarding the higher‐order photon‐number statistics that our PNR detection scheme resolves. While both reservoirs reconstruct monotonic functions with comparable accuracy, their performance diverges markedly for the strongly nonlinear targets, the quartic and damped sinusoidal functions, where $R^{2}$ increases from 0.69 in the classical case to 0.94 once the full Fock‐state distribution is retained. The complete function‐by‐function comparison is reported in Table S2. These results indicate that the nonclassical, higher‐order photon‐number correlations captured by our reservoir constitute a computational resource. By enriching the highly interconnected, high‐dimensional feature space in which the reservoir encodes information, they provide it with the nonlinearity needed to generalize across multiple function types of different complexity. In Note S7, we further discuss that this behavior is robust against the two dominant sources of noise in our implementation: the finite efficiency and dark counts of our single‐photon detectors, as well as the OAM–photon‐number coupling introduced by the multimode network. For the sake of completeness, we have included in Note S7, especially in Table S1, how our platform and performed tasks compare with other state‐of‐the‐art photonic platforms for quantum reservoir computing [54, 55, 56].

Scalability and fragility have long represented two major challenges to the practical realization of quantum technologies. Hybrid approaches that combine quantum measurements with classical physical platforms provide a promising route for overcoming these limitations and for developing quantum technologies that are simultaneously robust and scalable [57, 58, 59, 60]. Our results show that quantum advantages can be accessed by distilling multiparticle quantum systems from intrinsically noisy classical optical fields and processing them within a multidimensional photonic reservoir [28, 29, 61]. This capability broadens the possibilities for implementing quantum‐information protocols in realistic photonic platforms operating in the presence of noise and loss. The large photon fluxes available from classical light sources facilitate access to multiparticle systems, but the ultimate scalability of this class of hybrid quantum technologies remains linked to the capabilities of photon‐number‐resolving detection. In particular, accessing increasingly large multiparticle systems requires reliable Fock‐state projective measurements at progressively higher photon numbers. This limitation can be alleviated through detector multiplexing or by increasing the number of PNR detection channels, thereby extending the scale of experimentally accessible quantum systems. An additional practical constraint arises from the post‐selection of large multiparticle components. Although the high photon flux supplied by classical sources increases the rate at which these components can be accessed, the finite dead time and count‐rate capabilities of current PNR detection technologies can ultimately limit the rate of multiparticle distillation [14, 28, 35, 48]. We quantitatively examine these detector and post‐selection constraints, together with their implications for scalable quantum information processing, in Notes S5 and S6, with the measurement‐time tradeoff shown in Figure S3.

In conclusion, our demonstration of robust multiphoton quantum reservoirs presents a promising approach to overcoming the inherent fragility of current quantum technologies for information processing [1, 2, 3, 16]. This breakthrough was achieved by extracting bosonic or fermionic quantum multiphoton subsystems from classical thermal wavepackets [28, 29, 44]. Interestingly, this process allows us to engineer multidimensional quantum reservoirs hosting up to forty photons [14, 35]. Our PNR measurements enabled us to introduce nonlinearities that are essential for quantum reservoir computing [17, 18, 20]. Notably, isolating the vacuum dynamics of our reservoir enables the transformation of classical information processing routines into their quantum counterparts [29]. This unique platform was exploited to perform quantum simulations of many‐body thermodynamics in synthetic lattices, demonstrating its capacity to simulate nonlinear phenomena. Remarkably, we employed the complexity of our multiphoton quantum reservoir, consisting of eight hundred sixty‐one components, for the quantum prediction of nontrivial mathematical functions. These results highlight the robustness and scalability of hybrid quantum technologies against decoherence, particularly those combining classical light sources with nonclassical multiphoton detection [28, 29, 30].

## Conflicts of Interest

The authors declare no conflicts of interest.

## Supporting information


**Supporting File**: advs77225‐sup‐0001‐SuppMat.pdf.

## Data Availability

The data sets generated and/or analyzed during this study are available from the corresponding author or last author on reasonable request.
