# Supplementary material for: Multiphoton Quantum Reservoirs For Robust Multidimensional Computing
Source: Adv Sci (Weinh). 2026 Aug 19;13(59):e77225. doi: 10.1002/advs.77225 (PMC13487868; doi:10.1002/advs.77225)
Supplement: Supplementary file 1 [file ADVS-13-e77225-s001.pdf]

Advanced Science

WILEY

SUPPORTING INFORMATION

Supplementary Information - Multiphoton Quantum Reservoirs for Robust Multidimensional Computing

Mingyuan Hong[1] | Mario A. Quiroz-Juarez[2] | Riley B. Dawkins[1] | Fatemeh Mostafavi[1] | Jannatul Ferdous[1] | Addison Wilberg[1] | Blas M. Rodriguez-Lara[3] | Justin Woodring[1,4] | Chenglong You[1] | Roberto de J. León-Montiel[5] | Omar S. Magaña-Loaiza[1]

[1]Quantum Photonics Laboratory, Department of Physics & Astronomy, Louisiana State University, Baton Rouge, LA 70803, USA | [2]Centro de Física Aplicada y Tecnología Avanzadas, Universidad Nacional Autónoma de México, Querétaro 76230, Mexico | [3]Universidad Politécnica de Pachuca. Carr. Pachuca-Cd. Sahagún Km.20, Ex-Hda. Santa Bárbara. Zempoala, 43830 Hidalgo, Mexico | [4]School of Electrical Engineering and Computer Science, Louisiana State University, Baton Rouge, LA 70803, USA [5]Instituto de Ciencias Nucleares, Universidad Nacional Autónoma de México, Apartado Postal 70-543, 04510 Cd. Mx., México |

Correspondence: Mingyuan Hong (mingyuan@uestc.edu.cn)

Received: 30 April 2026 | **Revised:** 22 July 2026

Keywords: Multiphoton States | Quantum Simulation | Quantum Reservoir Computing | Photonic Lattices | Photonic Networks

ABSTRACT

This document provides supplementary information for “Multiphoton Quantum Reservoirs for Robust Multidimensional Computing.” We provide here the derivations for all formulas used in the main paper, as well as some further discussion of their interesting features. We also provide details of our experiment.

1 | Note 1: the Multidimensional Reservoir Network

The multidimensional reservoir network utilized in this paper involves the random unitary propagation of photonic modes with varying polarization and orbital angular momentum (OAM). Specifically, the annihilation operators associated with these modes are given by

$$\hat{a}_{s,\ell,p} = \int \frac{d^2x}{2\pi} \mathrm{LG}_p^\ell(\mathbf{x}) \hat{a}_s(\mathbf{x}), \tag{S1}$$

where

$$\mathrm{LG}_p^\ell(\mathbf{x}) = \sqrt{\frac{2p!}{\pi(p+|\ell|)!}} \frac{1}{w_0} \left(\frac{r\sqrt{2}}{w_0}\right)^{|\ell|} \exp\left(-\frac{x^2}{w_0^2}\right) L_p^{|\ell|}\left(\frac{2x^2}{w_0^2}\right) \exp(i\ell\phi), \tag{S2}$$

with $\mathbf{x} = (x, \phi)$ being the transverse polar coordinates of the light source. The $L_p^{|\ell|}$ functions are generalized Laguerre polynomials. Here, the subscript s corresponds to the polarization mode of the photons annihilated by this operator, and its value is either H or V. The subscript ℓ is the azimuthal index, and its value can be any integer (positive, negative, or zero). Lastly, the subscript p is the radial index, and its value can be any integer greater than or equal to 0. For a given input, each layer of the reservoir network applies a random unitary transformation over the mode space spanned by these $\hat{a}_{s,\ell,p}$. For simplicity of notation, we introduce the multi-index $\lambda \equiv (s, \ell, p)$ to represent one choice of a polarized OAM mode. With this, it is then possible to represent the action of the ith layer of the reservoir network with the unitary matrix

$$\hat{U}_i = \exp\left[\frac{1}{2}\sum_{\lambda\neq\lambda'}\left(\eta^{i*}_{\lambda\lambda'}\hat{a}_\lambda\hat{a}^\dagger_{\lambda'} - \eta^i_{\lambda\lambda'}\hat{a}_{\lambda'}\hat{a}^\dagger_\lambda\right)\right], \tag{S3}$$

where here the $1/2$ is employed to prevent double-counting and $\eta^i_{\lambda\lambda'}$ is the beam-splitter coefficient between the λ and λ' modes. This transformation represents the generalized beam-splitter transformation which is performed by the reservoir network. The total

unitary operator is then given by the produce of all layers' unitary operators. So, if the network has N layers, then the corresponding transformation for the reservoir is given by

$$\hat{U} = \prod_{i=1}^{N} \hat{U}_i = \prod_{i=1}^{N} \exp\left[\frac{1}{2} \sum_{\lambda \neq \lambda'} \left(\eta^{i*}_{\lambda\lambda'} \hat{a}_\lambda \hat{a}^\dagger_{\lambda'} - \eta^{i}_{\lambda\lambda'} \hat{a}_{\lambda'} \hat{a}^\dagger_{\lambda} \right) \right]. \tag{S4}$$

As such, the reservoir is completely characterized by the three-index tensor $\eta^i_{\lambda\lambda'}$, which can be represented by a vector of matrices

$$\vec{\boldsymbol{\eta}} = \bigoplus_{i=1}^{N} \boldsymbol{\eta}^i = \bigoplus_{i=1}^{N} \sum_{\lambda \neq \lambda'} \eta^i_{\lambda\lambda'} e^{\lambda} \otimes e^{\lambda'}, \tag{S5}$$

where the e^λ are the orthonormal basis vectors of the chosen matrix representation (and, as such, are only notationally-relevant). For a system of this type, we are generally interested in finding the most efficient way to compute the probability of observing a particular output for a given input. Such a task is exactly analogous to the Gaussian bosonic sampling problem, whose computational complexity is known to be $\#P$-complete [S1]. Specifically, it involves computing the permanent of a submatrix of $\hat{U}$. The size of this submatrix is related to the number of input and output modes in consideration, and as such, quantum advantages would only be expected for sufficiently-many input and output modes. Now, for practical applications it will be beneficial to have a description of this unitary transformation in the Fock-basis representation. To determine this, we start by noting that the unitary transformation $\hat{U}$ can equivalently be written in the form

$$\hat{U} \equiv \exp\left[\frac{1}{2} \sum_{\lambda \neq \lambda'} \left(\theta^{*}_{\lambda\lambda'} \hat{a}_\lambda \hat{a}^\dagger_{\lambda'} - \theta_{\lambda\lambda'} \hat{a}_{\lambda'} \hat{a}^\dagger_{\lambda} \right) \right], \tag{S6}$$

where here the $\theta_{\lambda,\lambda'}$ coefficients can be derived numerically from the $\vec{\boldsymbol{\eta}}$ tensor which corresponds to the experimental reservoir computing setup. Expressing this transformation in the Fock basis will additionally require a number of numerical computations. Specifically, we must first determine how the creation operators $\hat{a}^\dagger_\lambda$ evolve under the transformation $\hat{U}\hat{a}^\dagger_\lambda\hat{U}^\dagger$. To accomplish this task, we use the Baker-Campbell-Hausdorff formula, which states that

$$e^{-\hat{A}} \hat{B} e^{\hat{A}} = \sum_{n=0}^{\infty} \frac{1}{n!} C^{(n)}_{\hat{A}}[\hat{B}], \tag{S7}$$

where here we have defined

$$\begin{aligned} C^{(n)}_{\hat{A}}[\hat{B}] &= [C^{(n-1)}_{\hat{A}}[\hat{B}], \hat{A}], \\ C^{(0)}_{\hat{A}}[\hat{B}] &= \hat{B}. \end{aligned} \tag{S8}$$

In our case, we have that $\hat{B} \equiv \hat{a}^\dagger_\lambda$ and that $\hat{A} \equiv -\frac{1}{2} \sum_{\lambda \neq \lambda'} \left(\theta^{*}_{\lambda\lambda'} \hat{a}_\lambda \hat{a}^\dagger_{\lambda'} - \theta_{\lambda\lambda'} \hat{a}_{\lambda'} \hat{a}^\dagger_{\lambda} \right)$. Utilizing the canonical commutation relations for the $\hat{a}^\dagger_\lambda$ operators, we obtain

$$C^{(n)}_{\hat{A}}[\hat{a}^\dagger_\lambda] = \frac{1}{2} \sum_{\lambda_1 \neq \lambda} (\theta^*_{\lambda\lambda_1} - \theta_{\lambda_1\lambda}) \sum_{\lambda_2 \neq \lambda_1} (\theta^*_{\lambda_1\lambda_2} - \theta_{\lambda_2\lambda_1}) \dots \sum_{\lambda_n \neq \lambda_{n-1}} (\theta^*_{\lambda_{n-1}\lambda_n} - \theta_{\lambda_n\lambda_{n-1}}) \hat{a}^\dagger_{\lambda_n}. \tag{S9}$$

Analytically evaluating the coefficients in the full expansion of $\hat{U}\hat{a}^\dagger_\lambda\hat{U}^\dagger$ would be implausible, but these summations can be numerically approximated, yielding the expansion

$$\hat{U}\hat{a}^\dagger_\lambda\hat{U}^\dagger \equiv \sum_{\lambda'} u_{\lambda\lambda'} \hat{a}^\dagger_{\lambda'}, \tag{S10}$$

where $u_{\lambda\lambda'}$ are the coefficients obtained by grouping the $\hat{a}^\dagger_{\lambda'}$ terms in the expansion from Eq. (S7). Notably, this is the same numerical approach which is necessary to compute the $\theta_{\lambda\lambda'}$ from $\vec{\boldsymbol{\eta}}$. Utilizing this expansion, we will be able to calculate the Fock representation of $\hat{U}$. Specifically, we represent a Fock vector by $|\mathbf{n}\rangle$, where here $\mathbf{n} \equiv (n_{\lambda_1}, n_{\lambda_2}, \dots)$ denotes the vector of photon-number occupations in each mode. Notably, the components of $\hat{U}$ corresponding to finitely-many total quanta will be the only physically-relevant components, since any true, physical quantum state's Fock representation will have nonzero coefficients in the finite-quanta components alone. Now, using our above expression for how $\hat{U}$ acts on the individual creation operators, we obtain the nested multi-index expansion

$$\begin{aligned} \langle \mathbf{m} | \hat{U} | \mathbf{n} \rangle &= \langle \mathbf{m} | \prod_{n_\lambda \in \mathbf{n}} \frac{(\hat{U}\hat{a}^\dagger_\lambda\hat{U}^\dagger)^{n_\lambda}}{\sqrt{n_\lambda!}} \hat{U} |0\rangle \\ &= \langle \mathbf{m} | \prod_{n_\lambda \in \mathbf{n}} \frac{1}{\sqrt{n_\lambda!}} \sum_{I_{n_\lambda}} \prod_{\lambda' \in I_{n_\lambda}} u_{\lambda\lambda'} \hat{a}^\dagger_{\lambda'} |0\rangle \\ &= \langle \mathbf{m} | \sum_{I_\mathbf{n}} \prod_{I_{n_\lambda} \in I_\mathbf{n}} \frac{1}{\sqrt{n_\lambda!}} \prod_{\lambda' \in I_{n_\lambda}} u_{\lambda\lambda'} \hat{a}^\dagger_{\lambda'} |0\rangle \\ &= \sum_{\substack{I_\mathbf{n} \\ \forall \lambda'', \lambda''[I_\mathbf{n}] = m_{\lambda''}}} \left(\prod_{\lambda} \sqrt{\frac{m_\lambda!}{n_\lambda!}} \right) \left(\prod_{I_{n_\lambda} \in I_\mathbf{n}} \prod_{\lambda' \in I_{n_\lambda}} u_{\lambda\lambda'} \right) \\ &\equiv \gamma_{\mathbf{n},\mathbf{m}}(\vec{\boldsymbol{\eta}}). \end{aligned} \tag{S11}$$

Here, we have defined a number of multi-indices at each step to evaluate this product. First, on the second step, we have introduced the multi-index I_{n_λ}. Each multi-index I_{n_λ} represents a sequence of n_λ modes λ', and the sum over this multi-index is, therefore, over all such sequences. In the third step, we have then introduced the multi-index $I_{\mathbf{n}} \equiv (I_{n_{\lambda_1}}, I_{n_{\lambda_2}}, ...)$ of all such multi-indices I_{n_λ} which correspond to the n_λ in the vector $\mathbf{n}$. Then, in the fourth step, we have introduced the combinatorial function $\lambda''[I_{\mathbf{n}}]$, which counts the total number of times λ'' occurs throughout all of the $I_{n_\lambda} \in I_{\mathbf{n}}$. Finally, we have denoted this expression as $\gamma_{\mathbf{n},\mathbf{m}}(\vec{\eta})$ for simplicity. With this, we are able to write

$$\hat{U} = \sum_{\mathbf{n},\mathbf{m}} \gamma_{\mathbf{n},\mathbf{m}}(\vec{\eta})|\mathbf{n}\rangle\langle\mathbf{m}|. \tag{S12}$$

It is the classical computation of these parameters, $\gamma_{\mathbf{n},\mathbf{m}}(\vec{\eta})$, which is equivalent to the computation of the permanent of a submatrix of $\hat{U}$. By post-selecting on particular Fock-basis elements, however, we are able to measure them directly. Now, as mentioned previously, the computational difficulty of the $\#P$-complete $\gamma_{\mathbf{n},\mathbf{m}}(\vec{\eta})$ coefficients will depend on how many input and output modes are present in a given measurement. For our case specifically, the number of input modes is theoretically infinite, and therefore the classical difficulty of computing the $\gamma_{\mathbf{n},\mathbf{m}}(\vec{\eta})$ coefficients will depend entirely on how many output modes we choose to observe in a given measurement. The number of input modes is infinite because the input state of our system is given by Quantum Gaussian-Schell (QGS) light [S2]. In particular, our input source consists of two orthogonally-polarized QGS sources which exist in a mixed state. This light source's quantum state is given by a functional integral over random spatial profiles $\Sigma(\mathbf{x})$ as

$$\hat{\rho}_{\text{QGS}} = \bigotimes_s \int d\Sigma |\alpha_s\rangle\langle\alpha_s|_{s,\Sigma}, \tag{S13}$$

where the α_s are some parameters corresponding to the intensities of the individual sources and where $|\alpha_s\rangle\langle\alpha_s|_{s,\Sigma}$ is a coherent state composed of the modes

$$\hat{a}_{s,\Sigma} = \int \frac{d^2x}{(2\pi)^{3/2}} \Sigma(\mathbf{x})\hat{a}_s(\mathbf{x}). \tag{S14}$$

To write this state in the basis of orbital angular momentum, one must use the inherent Gaussian statistics of the functional integral over Σ. Specifically, these statistics are determined by the average of the electric field amplitudes over Σ, where $\langle E_s^{(+)}(\mathbf{x})\rangle_\Sigma = 0$ and where

$$\langle E_s^{(-)}(\mathbf{x})E_s^{(+)}(\mathbf{x}')\rangle_\Sigma = \sqrt{\bar{n}_s(\mathbf{x})\bar{n}_s(\mathbf{x}')}\,\mathrm{g}(\mathbf{x}-\mathbf{x}'), \tag{S15}$$

where here we have that $\bar{n}_s(\mathbf{x}) \propto \exp\left[-|\mathbf{x}|^2/\sigma_0\right]$, and that $\mathrm{g}(\mathbf{x}-\mathbf{x}') \propto \exp\left[-|\mathbf{x}-\mathbf{x}'|^2/\sigma_1\right]$ for some $\sigma_0, \sigma_1 > 0$. Then, to determine the distribution over λ modes of Σ, we introduce the complex amplitudes

$$E_\lambda^{(+)} \equiv E_{s,\ell,p}^{(+)} = \int d^2x \mathrm{LG}_p^{*\ell}(\mathbf{x})E_s^{(+)}(\mathbf{x}), \tag{S16}$$

where here $\lambda \equiv (s, \ell, p)$ as above. The statistical distribution of these amplitudes corresponds to the statistical distribution of coherent states in the (s, ℓ, p) mode of the QGS state [S3]. Since these amplitudes obey complex-Gaussian statistics, and since their mean is 0 for our source in particular, the statistics of our source will be completely defined by the covariance matrix

$$\Gamma_{\lambda\lambda'} = \langle E_\lambda^{(-)}E_{\lambda'}^{(+)}\rangle_\Sigma = \int d\mathbf{x}d\mathbf{x}'\mathrm{LG}_p^{\ell}(\mathbf{x})\mathrm{LG}_{p'}^{*\ell'}(\mathbf{x}')\sqrt{\bar{n}_s(\mathbf{x})\bar{n}_s(\mathbf{x}')}\,\mathrm{g}(\mathbf{x}-\mathbf{x}')\delta_{ss'}, \tag{S17}$$

where here we have $\lambda \equiv (s, \ell, p), \lambda' \equiv (s', \ell', p')$. The statistical distribution over these amplitudes, given by

$$P(\boldsymbol{\alpha}) = \frac{1}{4\pi^2\sqrt{|\boldsymbol{\Gamma}|}} e^{-\frac{1}{2}\boldsymbol{\alpha}^T\boldsymbol{\Gamma}^{-1}\boldsymbol{\alpha}} \tag{S18}$$

for $\boldsymbol{\alpha}$ the vector of real and imaginary parts of the coherent amplitudes $E_\lambda^{(+)}$ and for $|\cdot|$ the determinant operation, will then correspond directly to the quantum P-function of the state. As such, the total state in the OAM basis is given by

$$\hat{\rho}_{\text{QGS}} = \int d^2\boldsymbol{\alpha} P(\boldsymbol{\alpha})|\boldsymbol{\alpha}\rangle\langle\boldsymbol{\alpha}|, \tag{S19}$$

where $|\boldsymbol{\alpha}\rangle$ is the coherent state whose amplitudes in each λ mode correspond to the classical random variable $E_\lambda^{(+)}$. The covariance matrix $\Gamma_{\lambda\lambda'}$ is nonzero along its diagonal, and this means that the input state will have a non-zero population in each polarization-OAM mode. This is why our circuit has a theoretically-infinite number of input degrees of freedom.

---

2 | Note 2: Extracting quantum information from classical multiphoton reservoirs

In this section, we demonstrate how the theoretical framework of the above sections applies to our particular experimental realization. Recall that the quantum state which we are utilizing in our experiment is described by the QGS model as

$$\hat{\rho}_{\mathrm{QGS}} = \int d^2\boldsymbol{\alpha} P(\boldsymbol{\alpha}) |\boldsymbol{\alpha}\rangle\langle\boldsymbol{\alpha}|, \tag{S20}$$

where $P(\boldsymbol{\alpha})$ is the Gaussian probability density between the different OAM and polarization modes. Since our state has two modes with different polarizations, the input state can be re-written in the form

$$\hat{\rho}_{\mathrm{QGS}} = \int d^2\boldsymbol{\alpha}_H d^2\boldsymbol{\alpha}_V P_H(\boldsymbol{\alpha}_H) P_V(\boldsymbol{\alpha}_V) \left[\bigotimes_{\ell,p} |\alpha_{H,\ell,p}\rangle\langle\alpha_{H,\ell,p}|_{H,\ell,p} \right] \otimes \left[\bigotimes_{\ell',p'} |\alpha_{V,\ell',p'}\rangle\langle\alpha_{V,\ell',p'}|_{V,\ell',p'} \right]. \tag{S21}$$

Here, we have split up the integration variables into their H and V polarization components, and separated the P-function into its H and V polarization components as well. Now, each step $\hat{U}_i$ in our random unitary circuit $\hat{U}$ is composed of a number of waveplates and q-plates. The Jones matrix corresponding to a general waveplate is given, in the (H, V) basis, by

$$\mathbf{J}_{\mathrm{WP}}(\theta, \eta) = \begin{pmatrix} c_\theta^2 + e^{i\eta} s_\theta^2 & (1 - e^{i\eta}) c_\theta s_\theta \\ (1 - e^{i\eta}) c_\theta s_\theta & s_\theta^2 + e^{i\eta} c_\theta^2 \end{pmatrix}, \tag{S22}$$

where we are using the shorthands $c_\theta \equiv \cos(\theta)$ and $s_\theta \equiv \sin(\theta)$. Moreover, θ is the waveplate's angle and η is a parameter which corresponds to the ratio between the wavelength ω_0 of our source and the paraxial length of the waveplate. For a half-waveplate (HWP), we have $\eta = \pi$, and for a quarter-waveplate (QWP), we have $\eta = \pi/2$. For a given choice of OAM (ℓ, p), the action of a HWP $\hat{U}_{\mathrm{HWP}}$ is then given by the unitary transformation

$$\begin{aligned} \hat{U}_{\mathrm{HWP}} \hat{a}_{H,\ell,p} \hat{U}_{\mathrm{HWP}}^\dagger &= c_{2\theta} \hat{a}_{H,\ell,p} + s_{2\theta} \hat{a}_{V,\ell,p}, \\ \hat{U}_{\mathrm{HWP}} \hat{a}_{V,\ell,p} \hat{U}_{\mathrm{HWP}}^\dagger &= s_{2\theta} \hat{a}_{H,\ell,p} - c_{2\theta} \hat{a}_{V,\ell,p}. \end{aligned} \tag{S23}$$

Similarly, the unitary transformation associated with a QWP $\hat{U}_{\mathrm{QWP}}$ is given by

$$\begin{aligned} \hat{U}_{\mathrm{QWP}} \hat{a}_{H,\ell,p} \hat{U}_{\mathrm{QWP}}^\dagger &= (c_\theta^2 + i s_\theta^2) \hat{a}_{H,\ell,p} + (1 - i) c_\theta s_\theta \hat{a}_{V,\ell,p}, \\ \hat{U}_{\mathrm{QWP}} \hat{a}_{V,\ell,p} \hat{U}_{\mathrm{QWP}}^\dagger &= (1 - i) c_\theta s_\theta \hat{a}_{H,\ell,p} + (s_\theta^2 + i c_\theta^2) \hat{a}_{V,\ell,p}. \end{aligned} \tag{S24}$$

Luckily, since these transformations are well-known in terms of the Jones matrix formalism, we do not have to start from the exponential form of Eq. (S3) to find the unitary action of these waveplates on the individual annihilation operators. A q-plate performs a transformation which is very similar to a HWP, but with the addendum that it performs this operation on the right and left circular polarization basis and that it is a transformation between modes with different values of ℓ as opposed to modes with the same value of ℓ. Specifically, the Jones matrix of a q-plate is given by

$$\mathbf{J}_{q\text{-plate}}(\delta, q) = \begin{pmatrix} i c_{\delta/2} & s_{\delta/2} \\ s_{\delta/2} & i c_{\delta/2} \end{pmatrix}, \tag{S25}$$

where here q can be any integer multiple of $1/2$, and where δ is the phase retardation of the q-plate. The corresponding unitary action $\hat{U}_{q\text{-plate}}$ of this Jones matrix on annihilation operators is then given by

$$\begin{aligned} \hat{U}_{q\text{-plate}} \hat{a}_{L,\ell,p} \hat{U}_{q\text{-plate}}^\dagger &= i c_{\delta/2} \hat{a}_{L,\ell,p} + s_{\delta/2} \hat{a}_{R,\ell-2q,p}, \\ \hat{U}_{q\text{-plate}} \hat{a}_{R,\ell-2q,p} \hat{U}_{q\text{-plate}}^\dagger &= s_{\delta/2} \hat{a}_{L,\ell,p} + i c_{\delta/2} \hat{a}_{R,\ell-2q,p}. \end{aligned} \tag{S26}$$

Here, we see why q can only be integer multiples of $1/2$, since $2q$ is the change in OAM. q-plates with other values of q have a more complicated behavior, generating photons with a non-OAM phase structure. In our experiment, a single step of the circuit is given by a HWP at some angle θ, a QWP at some angle ϕ, and a q-plate with $\delta = \pi$ and $q = 1/2$. This general step transformation $\hat{U}_S(\theta, \phi)$ is then defined by the action

$$\begin{aligned} \hat{U}_S(\theta, \phi) \hat{a}_{L,\ell,p} \hat{U}_S^\dagger(\theta, \phi) &= e^{i\pi/4 + 2i\theta} \hat{a}_{L,\ell,p} + e^{-i\pi/4 - 2i(\theta - \phi)} \hat{a}_{R,\ell-1,p}, \\ \hat{U}_S(\theta, \phi) \hat{a}_{R,\ell-1,p} \hat{U}_S^\dagger(\theta, \phi) &= e^{i\pi/4 + 2i(\theta - \phi)} \hat{a}_{L,\ell,p} + e^{i\pi/4 - 2i\theta} \hat{a}_{R,\ell-1,p}. \end{aligned} \tag{S27}$$

For the quantum reservoir, we randomize θ and ϕ at each step. After N steps, with waveplate angles $\boldsymbol{\theta} = (\theta_1, ..., \theta_N), \boldsymbol{\phi} = (\phi_1, ..., \phi_N)$ and total unitary transformation $\hat{U}(\boldsymbol{\theta}, \boldsymbol{\phi})$, this transformation becomes

$$\begin{aligned} \hat{U}(\boldsymbol{\theta}, \boldsymbol{\phi}) \hat{a}_{L,\ell,p} \hat{U}^\dagger(\boldsymbol{\theta}, \boldsymbol{\phi}) &= \sum_{\ell'} \left(c_{LL,\ell\ell'}(\boldsymbol{\theta}, \boldsymbol{\phi}) \hat{a}_{L,\ell',p} + c_{LR,\ell\ell'}(\boldsymbol{\theta}, \boldsymbol{\phi}) \hat{a}_{R,\ell',p} \right), \\ \hat{U}(\boldsymbol{\theta}, \boldsymbol{\phi}) \hat{a}_{R,\ell-1,p} \hat{U}^\dagger(\boldsymbol{\theta}, \boldsymbol{\phi}) &= \sum_{\ell'} \left(c_{RL,\ell\ell'}(\boldsymbol{\theta}, \boldsymbol{\phi}) \hat{a}_{L,\ell',p} + c_{LR,\ell\ell'}(\boldsymbol{\theta}, \boldsymbol{\phi}) \hat{a}_{RR,\ell',p} \right). \end{aligned} \tag{S28}$$

Here, we have introduced the coefficients $c_{ss',\ell\ell'}(\theta,\phi)$, which can be computed numerically. Notably, the number of such components which are nonzero is linearly proportional to the number of steps in the circuit. From here, what's important is the effect on coherent state vectors. Specifically, we have that

$$\bigotimes_{s,\ell,p} \hat{U}(\theta,\phi)|\alpha_{s,\ell,p}\rangle_{s,\ell,p} = \bigotimes_{s,\ell,p} \left| \sum_{\ell',s'} c^*_{s's,\ell'\ell}(\theta,\phi)\alpha_{s',\ell',p} \right\rangle_{s,\ell,p} . \tag{S29}$$

The output state is then obtained by applying this transformation to Eq. (S21). We are then interested in the outcomes obtained by making a post-selective measurement over a particular value of ℓ and over a particular number of photons N. To obtain this, we first note that the distribution of the random variables

$$A_{s,\ell,p} = \sum_{\ell',s'} c^*_{s's,\ell'\ell}(\theta,\phi)\alpha_{s',\ell',p} \tag{S30}$$

is Gaussian, since it is the sum of Gaussian random variables. Furthermore, it has mean zero, and its covariance terms are given by

$$\langle A_{s,\ell,p}A^*_{s',\ell',p'}\rangle_\Sigma = \sum_{k,k',s''} c^*_{s''s,k\ell}(\theta,\phi)c_{s''s',k'\ell'}(\theta,\phi)\langle \alpha_{s,\ell,p}\alpha^*_{s',\ell',p'}\rangle_\Sigma. \tag{S31}$$

In our experiment, we only measure outputs where $p = 0$. Notably, in this case, we have that

$$\langle \alpha_{s,\ell,0}\alpha^*_{s',\ell',0}\rangle_\Sigma \approx \bar{n}_0 e^{-\frac{|\ell|^2+|\ell'|^2}{2\lambda}}\delta_{ss'} \tag{S32}$$

for some $\bar{n}_0 \geq 0$ and for some $\lambda \geq 0$. A measurement of photon-number for a given OAM value ℓ is then obtained by taking the expectation value of the operator

$$\hat{n}_\ell = \hat{a}^\dagger_{H,\ell,0}\hat{a}_{H,\ell,0} + \hat{a}^\dagger_{V,\ell,0}\hat{a}_{V,\ell,0}, \tag{S33}$$

where post-selective measurements on N photons can then be made via the operator

$$|N\rangle\langle N|_\ell =: \left(\frac{\hat{n}_\ell^N}{N!}e^{-\hat{n}_\ell}\right):, \tag{S34}$$

for $: \cdot :$ being the normal-ordering process. Finally, the outcome of this measurement is given by

$$\mathrm{Tr}[\hat{\rho}_{\mathrm{QGS}}|N\rangle\langle N|_\ell] = \int d^2A_{H,\ell,0}d^2A_{V,\ell,0}P(A_{H,\ell,0},A_{V,\ell,0})\left[\frac{\left(|A_{H,\ell,0}|^2+|A_{V,\ell,0}|^2\right)^N}{N!}e^{-|A_{H,\ell,0}|^2-|A_{V,\ell,0}|^2}\right], \tag{S35}$$

where $P(A_{H,\ell,0},A_{V,\ell,0})$ is the Gaussian distribution of the form given by Eq. (S18) for the two complex-Gaussian random variables $A_{H,\ell,0}$ and $A_{V,\ell,0}$. The evaluated form of this integral is, also, well-known [S3]. A much simpler case of this model is found in the single-input-mode random walk, where $\theta = \phi = 0$, and where the initial state is filtered so that only the $\ell = p = 0$ mode is present at the input. Specifically, the state at the input in this case is given by

$$\hat{\rho}_{in} = \left(\int d^2\alpha_H P_H(\alpha_H)|\alpha_H\rangle\langle\alpha_H|_{H,0,0}\right) \otimes \left(\int d^2\alpha_V P_V(\alpha_V)|\alpha_V\rangle\langle\alpha_V|_{V,0,0}\right), \tag{S36}$$

and the coherent-state transformation is given by

$$\bigotimes_{s} \hat{U}(0,0)|\alpha_s\rangle_{s,0,0} = \bigotimes_{s,\ell} \left| \sum_{s'} c^*_{s's,0\ell}(0,0)\alpha_{s',0,0} \right\rangle_{s,\ell,0} . \tag{S37}$$

From here, we would then define $A_{s,\ell,0}$ terms in exactly the same manner as before and evaluate Fock projections according to the analogous Eq. (S35). These measurements enable us to extract quantum information from classical multiphoton reservoirs. These two examples showcase the utility of a thermal input state, through which the random-walk and quantum reservoir unitary transformations act nicely, and in such a way that the output statistics can be predicted simply and without reference to the complicated Eq. (S11). The version of this computation with a full QGS input source also provides us with one example of an infinite-mode input configuration for the reservoir, the computability of which would be classically difficult without access to the state's Gaussian properties, but which can be readily computed via our quantum reservoir.

3 | Note 3: The Thermalization and Antithermalization Hamiltonians

As a consequence of the large unitary transformation parameterized by $\vec{\eta}$, a number of interesting statistical behaviors can be observed within the context of our reservoir quantum computer. Specifically, when we focus on subsystems at specific steps in the reservoir quantum computer, we observe that their multiphoton statistics are changing. These statistics are simulated via projections onto multiple modes simultaneously (for instance, we might collect photons from all modes with a particular ℓ value at a single detector). The unitary transformation $\hat{U}$ of our quantum reservoir causes the H and V portions of the input state to undergo distinct transformations in the OAM basis. As such, a given OAM projection might yield different measurement statistics at different points in the reservoir, depending on how much of the source at that step is originating from each polarization mode. In the regime of low-mean-photon-number, these measured statistics are known to mimic those of a single-mode displaced thermal state, whose P-function representation is given by

$$\hat{\rho}_{\bar{n},\mu} = \int d^2\alpha \frac{1}{\pi\bar{n}} \exp\left[-\frac{|\alpha-\mu|^2}{\bar{n}}\right] |\alpha\rangle\langle\alpha|. \tag{S38}$$

Here, $\bar{n}$ is the thermal amplitude of these equivalent statistics and μ is the coherent amplitude. The effect of $\hat{U}$ on these statistics can be represented by a change in the ratio between $\bar{n}$ and $|\mu|^2$. To see why, note that

$$\begin{aligned} \mathrm{Tr}\left[\hat{\rho}_{\bar{n},\mu}\hat{a}^\dagger\hat{a}\right] &= \bar{n} + |\mu|^2, \\ \mathrm{Tr}\left[\hat{\rho}_{\bar{n},\mu}\hat{a}^\dagger\hat{a}^\dagger\hat{a}\hat{a}\right] &= 2\bar{n}^2 + 4\bar{n}|\mu|^2 + |\mu|^4. \end{aligned} \tag{S39}$$

As such, the coherence of the state is given by

$$g^{(2)}(0) = 2 - \frac{|\mu|^4}{(\bar{n}+|\mu|^2)^2}. \tag{S40}$$

So, if $|\mu|^2 \gg \bar{n}$, the state will be coherent, and if $|\mu|^2 \ll \bar{n}$ the state will be thermal. The Hamiltonian which causes this ratio to change is given by

$$\hat{H} = \left(\omega_0 + i\frac{\dot{A}^*}{A^*}\right)\hat{a}^\dagger\hat{a} + i\left(\dot{B} - B\frac{\dot{A}^*}{A^*} + i\omega_0 B\right)\hat{a}^\dagger - i\left(\dot{B}^* - B^*\frac{\dot{A}}{A} + i\omega_0 B^*\right)\hat{a}, \tag{S41}$$

where ω_0 is the frequency of the collected photons, $A(t)$ is a function which represents the change in thermal amplitude, and $B(t)$ is a function which represents the change in coherent amplitude. To see how the state evolves under this effective Hamiltonian description, we use Heisenberg's equations of time-evolution to get

$$\dot{\hat{a}}(t) = -i[\hat{a},\hat{H}] = \left(\frac{\dot{A}^*}{A^*} - i\omega_0\right)\hat{a} + \left(\dot{B} - B\frac{\dot{A}^*}{A^*} + i\omega_0 B\right). \tag{S42}$$

The solution of this differential equation is then given by

$$\hat{a}(t) = A^*(t)e^{-i\omega_0 t}\hat{a} + B(t). \tag{S43}$$

The effect of this transformation on coherent states is well-known to be

$$|\alpha\rangle \to |A(t)e^{i\omega_0 t}\alpha + B(t)\rangle, \tag{S44}$$

and applying this to the state $\hat{\rho}_{\bar{n},\mu}$, then performing an appropriate coordinate transformation, yields the evolved state

$$\hat{\rho}_{\bar{n},\mu}(t) = \int d^2\alpha \frac{1}{\pi|A(t)|^2\bar{n}} \exp\left[-\frac{|\alpha - \mu A(t)e^{i\omega_0 t} - B(t)|^2}{\bar{n}|A(t)|^2}\right] |\alpha\rangle\langle\alpha|. \tag{S45}$$

This new state will have second-order coherence given by, at time t,

$$g^{(2)}(0) = 2 - \frac{\left|\mu + \frac{B(t)}{A(t)}e^{-i\omega_0 t}\right|^4}{\left(\bar{n} + \left|\mu + \frac{B(t)}{A(t)}e^{-i\omega_0 t}\right|^2\right)^2}. \tag{S46}$$

So, we can see that, notably, the coherence statistics of the source will only change if $B(t)$ is nonzero. Depending on our choice of $A(t)$ and $B(t)$, this Hamiltonian can correspond to either thermalization or antithermalization of the observed statistics. This result shows that our platform is capable of simulating Hamiltonians of the form given by $\hat{H}$, something which can be useful for a variety of applications.

4 | Note 4: Details of Experiment

In this section, we describe the experimental implementation of the multidimensional photonic reservoir. Specifically, we describe the preparation of sources, the configuration of the optical network, and the detection methods employed to analyze the quantum dynamics presented in the main text.

The optical sources utilized in our experiments comprise a coherent light source produced by a laser at 633 nm, and a thermal source generated by passing the coherent laser beam through a rotating ground glass [S4]. Collection of both coherent and thermal light is performed using single-mode fibers. However, for the measurement of the classical random walk depicted in Figure 2d, a multimode fiber is used instead.

To prepare the initial multimode input state $|H, \ell = 0\rangle_1 + |V, \ell = 0\rangle_2$ as depicted in Figure 3, we independently generate two thermal wavepackets with horizontal (H) and vertical (V) polarizations. These wavepackets are subsequently combined using a 2×2 fiber beam splitter, yielding the desired input state.

The generated optical beam then follows a common optical path. The light exiting the fiber is first collimated by a lens (f = 25.4 mm) and subsequently directed into a 4-f optical system composed of two lenses with focal lengths of 150 mm and 200 mm. Following this 4-f system, a polarizer selects the horizontally polarized beam. A half-wave plate (HWP) then rotates the polarization, which enables the polarization to serve as a degree of freedom for the quantum reservoir. For the specific experiment depicted in Figure 3, the polarizer is omitted to preserve both polarizations. To generate left- and right-circularly polarized light for the input to the q-plates, a quarter-wave plate (QWP) is used, converting linear polarization into circular polarization.

Following the source preparation, the photons enter our designed photonic network, which consists of multiple half-wave plates (HWPs), quarter-wave plates (QWPs), and q-plates. Our experimental setup incorporates three q-plates with topological charge $q = 1/2$ and one q-plate with $q = 1$. Each q-plate alters the orbital angular momentum (OAM) of a photon by an amount of $\pm 2q$, depending on the incident polarization. Specifically, when a left-circularly polarized photon passes through a q-plate with $q = 1/2$, its OAM increases by 1, and its polarization state converts to right-circular polarization. Conversely, a photon with right-circular polarization experiences an OAM decrease of 1, and its polarization converts to left-circular polarization.

In our photonic network, we have used the following configuration: q-plate ($q = 1/2$) → HWP → QWP → q-plate ($q = 1/2$) → HWP → QWP → q-plate ($q = 1/2$) → HWP → QWP → q-plate ($q = 1$). As an example, for the quantum reservoir computing experiment, we rotate the half-wave plate placed before the photonic network from 0° to 45° in steps of 0.5°. This produces 91 distinct input states, which are used for training and testing in the reservoir computing task—enabling quantum predictions of nontrivial mathematical functions.

After exiting the photonic network, photons pass through another 4-f system consisting of two lenses (200 mm focal length). The beam is then split into two arms, each directed onto a spatial light modulator (SLM) for orbital angular momentum (OAM) measurement. The first diffraction order from each SLM is isolated using an additional 4-f system (150 mm lenses) with an iris placed at the Fourier plane. Finally, each beam is focused by lenses into polarization-maintaining single-mode fibers mounted on precision displacement stages. These fibers are coupled to avalanche photodiodes (APDs) connected to a time-tagger, allowing photon-number distribution measurements. The APDs are Excelitas SPCM-AQRH-14-FC modules and the time tagger is a PicoQuant MultiHarp 150. The time tagger was operated with an 80 ps temporal resolution. For PNR measurements, the time tags were grouped into 1 μs acquisition windows. A quantitative analysis of the fidelity and scalability of the time-binned photon-number-resolving measurement is provided in Supplementary Information Note 5, while the tradeoff between measurement time, post-selected photon number, and statistical precision is analyzed in Supplementary Information Note 6.

Although the same OAM–polarization platform and detection system are used throughout the experiments in Figures 2-4, the optical network is configured differently for the quantum random walk, the thermalization and anti-thermalization measurements, and quantum reservoir computing. These task-specific settings determine how the circular-polarization components are redistributed before entering the subsequent q-plates and therefore control the accessible OAM pathways.

For the quantum random walk measurements in Figure 2, the reservoir is operated in an ordered configuration. The input is restricted to the spatial mode $\ell = 0$, and an HWP is inserted between successive OAM-shifting operations to interchange the left- and right-circular polarization components. Since a q-plate simultaneously changes the OAM and flips the circular polarization, the following HWP flips the polarization back before the next q-plate. Consequently, each of the two OAM branches continues to evolve in the same direction in successive steps; after the xth nominal step, the two branches occupy the OAM components $\ell = +x$ and $\ell = -x$, as shown in Figure 2b. The OAM distribution at each step is measured by projecting the output field onto individual OAM modes with the SLM and reconstructing the corresponding photon-number distribution from the APD time tags. The seven-particle QRW is obtained by retaining the $n = 7$ outcomes for each measured OAM projection and normalizing the resulting counts over the measured OAM modes, yielding $P(\ell|n = 7)$. For the classical random walk in Figure 2d, a multimode fiber is used at the source stage, while the output is measured with the same OAM- and photon-number-resolved detection scheme. For the two-arm measurements in Figure 2e, the two SLMs independently project the output onto OAM modes in detection arms 1 and 2, and the joint OAM distributions are conditioned on the selected photon-number pair (n_1, n_2).

For the thermalization and anti-thermalization measurements in Figure 3, the input is the two-component thermal state described above, and both polarization components are preserved. In contrast to the ordered QRW configuration, both an HWP and a QWP are used between successive q-plates. Their combined polarization transformation generally prepares a superposition of left- and right-circular polarization before the next q-plate. The two circular components are then coupled to different OAM shifts, producing the richer set of OAM pathways and lattice nodes shown in Figure 3a. Photon-number distributions are measured at selected nodes of this synthetic OAM lattice. For each selected node, the joint photon-number distribution $P(n_1, n_2)$ is recorded and the corresponding marginal photon-number distributions are used to evaluate $g^{(2)}(0)$. Following different paths through the network therefore reveals

either an increase or a decrease in $g^{(2)}(0)$, corresponding to the thermalization and anti-thermalization dynamics reported in Figures 3b and 3c.
For the quantum reservoir computing measurements in Figure 4, the internal wave-plate angles are randomly chosen and then held fixed throughout the complete measurement so that all inputs are processed by the same physical reservoir. The input polarization is scanned from horizontal to vertical by rotating the HWP before the network from 0° to 45° in 0.5° steps, yielding 91 input states. For each input state, we measure 21 OAM projections, $\ell = -10, \ldots, 10$. Each polarization-OAM setting is acquired for 1 s and divided into 10^6 acquisition windows of 1 μs. The measured photon-number probabilities from $n = 0$ to $n = 40$ for all 21 OAM projections are assembled into an 861-component reservoir output vector. The random but fixed polarization-OAM coupling, together with the measurement-induced nonlinearity provided by photon-number projection, maps each polarization-encoded input onto a reproducible high-dimensional output distribution. The raw acquisition time for the complete reservoir-computing dataset is therefore 91 × 21 s = 1911 s, corresponding to approximately 32 min.
In Fig. S1, we present representative data samples obtained from the quantum reservoir computing experiment.

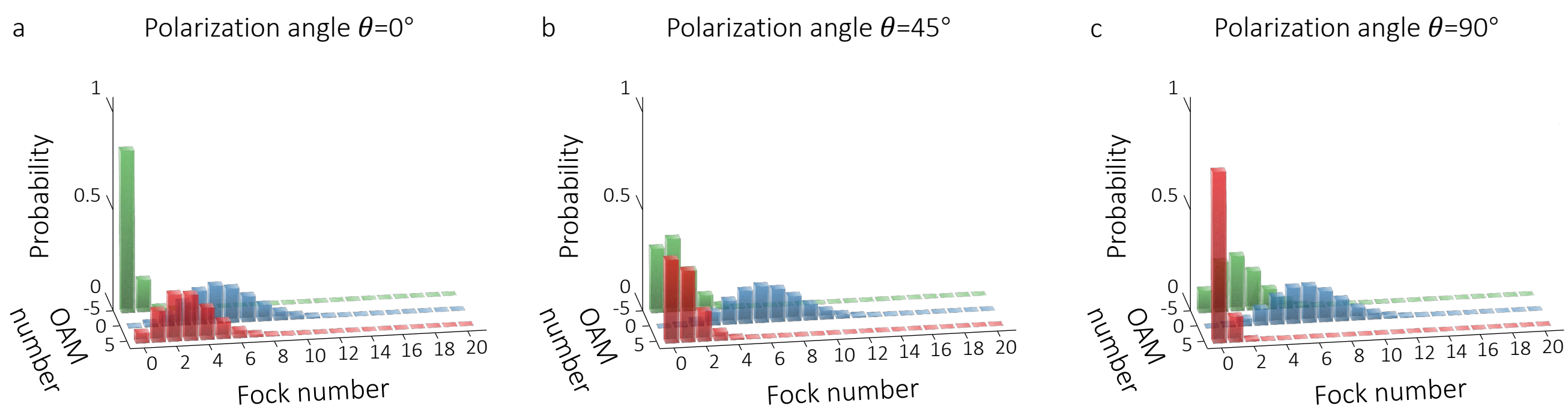

FIGURE S1 | **Samples of quantum reservoir data**. These three panels present our photon-number distributions at 0°, 45° and 90°, post-selecting on OAM = −5, 0, and 5. These data are further used in the quantum reservoir computing for the prediction of nontrivial mathematical functions.

5 | Note 5: Limitations and Scalability of Photon-Number-Resolving Detection

Photon-number projective measurements provide the nonlinear measurement readout used throughout our multiphoton quantum-information platform. In this section, we quantify the limitations of the time-binned photon-number-resolving measurement implemented with click detectors and compare its response with that of an ideal photon-number-resolving detector. To make this comparison transparent, we first establish the ideal thermal photon-number benchmark, then construct the time-binned click-detector model, and finally evaluate its fidelity and scalability.
We begin with the theoretical description of an ideal photon-number-resolving measurement. For simplicity, we assume that the detector collects the complete transverse profile of the optical field and define the detection time as the interval over which photons are absorbed and assigned to a single measurement outcome. For one polarization mode, the corresponding photon-number operator is

$$\hat{n} = \frac{1}{(2\pi)^3} \int_0^t dz \int d^2x \hat{a}^\dagger(\mathbf{r})\hat{a}(\mathbf{r}). \tag{S47}$$

Here, $\mathbf{r} \equiv (\mathbf{x}, z)$ denotes the three-dimensional position, with z the paraxial coordinate, t the measurement time, and $\hat{a}(\mathbf{r})$ the photon-density annihilation operator satisfying $[\hat{a}(\mathbf{r}), \hat{a}^\dagger(\mathbf{r}')] = (2\pi)^3\delta(\mathbf{r} - \mathbf{r}')$. The non-negative integer eigenvalues of $\hat{n}$ correspond to the possible photon-number outcomes. The POVM associated with detecting N photons is

$$\hat{n}_N =: \frac{\hat{n}^N}{N!} e^{-\hat{n}} :, \tag{S48}$$

where $:\cdot:$ denotes normal ordering. The photon-number operator may therefore be written as

$$\hat{n} = \sum_{N=0}^{\infty} N\hat{n}_N. \tag{S49}$$

We next specify the optical state against which the ideal and time-binned measurements will be compared. We consider a thermal field whose temporal coherence is sufficiently long that the state is effectively single-mode over the detection interval, with P-function

representation

$$\hat{\rho} = \int d^2\alpha \frac{1}{\pi\bar{n}} \exp\left[-\frac{|\alpha|^2}{\bar{n}}\right] |\alpha\rangle\langle\alpha|. \tag{S50}$$

Here, $\bar{n}$ is the mean photon number of the thermal state and $|\alpha\rangle$ is a coherent state defined by the annihilation operator

$$\hat{a} = \frac{1}{\sqrt{t\pi\epsilon^2}(2\pi)^{3/2}} \int_0^t dz \int_{B(0,\epsilon)} d^2x \hat{a}(\mathbf{r}), \tag{S51}$$

where $B(0,\epsilon)$ denotes a circle of radius ϵ centered at the origin. The normalization is chosen so that $[\hat{a}, \hat{a}^\dagger] = 1$. An ideal photon-number-resolving detector then yields the familiar thermal distribution

$$\mathrm{Tr}[\hat{\rho}\hat{n}_N] = \frac{\bar{n}^N}{(1+\bar{n})^{1+N}}. \tag{S52}$$

An ideal photon-number-resolving detector can, in principle, distinguish an arbitrary number of photons within a single detection interval t. Transition-edge sensors provide the closest current implementation and can resolve more than ten photons in one measurement. Our experiment instead uses commercially available click detectors in a time-binned photon-counting architecture. A click detector distinguishes a vacuum from a non-vacuum event but does not, in a single click measurement, identify the exact number of incident photons. Its measurement operator is

$$\hat{n}_C = \sum_{N=1}^{\infty} \hat{n}_N = \hat{\mathbb{I}} - \hat{n}_0. \tag{S53}$$

Here, $\hat{\mathbb{I}}$ is the identity operator. Although one click detector cannot distinguish different nonzero photon numbers in a single time interval, an effective approximation to photon-number-resolved detection can be obtained by dividing the total measurement time t into uniform subintervals of duration Δt and performing a click measurement in each subinterval. The interval Δt is chosen to exceed the detector timing jitter and recovery time while keeping the photon occupation of each subinterval sufficiently small. Summing the click outcomes across these subintervals then approximates the response of an ideal photon-number-resolving detector. We formalize this construction by defining the sub-number operators

$$\hat{n}(m) = \frac{1}{(2\pi)^3} \int_{m\Delta t}^{(m+1)\Delta t} dz \int d^2x \hat{a}^\dagger(\mathbf{r})\hat{a}(\mathbf{r}). \tag{S54}$$

Here, m is an integer ranging from 0 to $t/\Delta t - 1 = B - 1$, with B the total number of time bins. The corresponding POVMs are

$$\hat{n}_N(m) =: \frac{\hat{n}^N(m)}{N!} e^{-\hat{n}(m)} :, \tag{S55}$$

and the click operator for each temporal bin is

$$\hat{n}_C(m) = \hat{\mathbb{I}} - \hat{n}_0(m). \tag{S56}$$

The resulting binned number operator is

$$\hat{n}_{\mathrm{bin}} = \sum_m (\hat{\mathbb{I}} - \hat{n}_0(m)) = B \cdot \hat{\mathbb{I}} - \sum_m \hat{n}_0(m). \tag{S57}$$

If no vacuum event is recorded in any temporal bin, the measurement returns the maximal binned outcome B, whereas vacuum in every bin corresponds to an outcome of zero. Reliable operation therefore requires the number of bins B to remain sufficiently large compared with the mean photon number of the measured field. To obtain the operator associated with measuring a binned outcome N, we consider all measurement records in which N temporal bins contain one or more photons and the remaining $B - N$ bins contain vacuum. Summing over all choices of the N occupied bins gives

$$\hat{n}_{\mathrm{bin},N} = \sum_{\mathbf{M}} \prod_{m \in \mathbf{M}} (\hat{\mathbb{I}} - \hat{n}_0(m)) \prod_{m' \notin \mathbf{M}} \hat{n}_0(m'), \tag{S58}$$

where the multi-index $\mathbf{M}$ denotes a particular selection of N occupied bins.

To evaluate the response of this time-binned detector for the thermal benchmark introduced above, we express the optical mode as an equal superposition of B temporal modes,

$$\hat{a} = \sum_m \frac{1}{\sqrt{B}} \hat{a}_m, \tag{S59}$$

with

$$\hat{a}_m = \frac{1}{\sqrt{\Delta t\pi\epsilon^2}(2\pi)^{3/2}} \int_{m\Delta t}^{(m+1)\Delta t} dz \int_{B(0,\epsilon)} d^2x \hat{a}(\mathbf{r}). \tag{S60}$$

These operators satisfy $[\hat{a}_m, \hat{a}_{m'}^\dagger] = \delta_{mm'}$. The transformation maps $|\alpha\rangle$ onto the B-mode coherent state $|\alpha/\sqrt{B}\rangle_0 \otimes \cdots \otimes |\alpha/\sqrt{B}\rangle_{B-1}$. Applying this transformation to the thermal state and changing variables gives

$$\hat{\rho} = \int d^2\alpha \frac{1}{\pi(\bar{n}/B)} \exp\left[-\frac{|\alpha|^2}{(\bar{n}/B)}\right] \bigotimes_m |\alpha\rangle\langle\alpha|_m. \tag{S61}$$

Taking the trace with $\hat{n}_{\text{bin},N}$ then yields

$$\begin{aligned}
\mathrm{Tr}[\hat{\rho}\hat{n}_{\text{bin},N}] &= \int d^2\alpha \frac{1}{\pi(\bar{n}/B)} \exp\left[-\frac{|\alpha|^2}{(\bar{n}/B)}\right] \binom{B}{N} \left(1 - e^{-|\alpha|^2}\right)^N \left(e^{-|\alpha|^2}\right)^{B-N} \\
&= \frac{1}{\pi(\bar{n}/B)} \binom{B}{N} \int r dr d\theta \exp\left[-\frac{r^2}{(\bar{n}/B)}\right] \left(1 - e^{-r^2}\right)^N \left(e^{-r^2}\right)^{B-N} \\
&= \frac{2B}{\bar{n}} \binom{B}{N} \int_1^0 \left(-\frac{du}{2u}\right) u^{B-N+B/\bar{n}} (1-u)^N \\
&= \frac{B}{\bar{n}} \binom{B}{N} \int_0^1 du u^{B-N+B/\bar{n}-1} (1-u)^N \\
&= \frac{B}{\bar{n}} \binom{B}{N} \beta(B - N + B/\bar{n}, N+1) \\
&= \frac{B}{\bar{n}} \binom{B}{N} \frac{\Gamma(B - N + B/\bar{n})\Gamma(N+1)}{\Gamma(B + B/\bar{n} + 1)} \\
&= \frac{B}{\bar{n}} \left(\frac{B!}{(B-N)!}\right) \left(\frac{\Gamma(B - N + B/\bar{n})}{\Gamma(B + B/\bar{n} + 1)}\right).
\end{aligned} \tag{S62}$$

Here, $\beta(x, y)$ and $\Gamma(x)$ denote the beta and gamma functions, respectively. We quantify the performance of the time-binned measurement by comparing the distribution obtained for $B = 20$ with the corresponding ideal thermal photon-number distribution, as shown in Fig. S2**a**. The value $B = 20$ is the number of bins used in our detector model. This is because the detector's dead time is 50 ns, and a 1 μs measurement interval contains twenty 50 ns periods. For two probability distributions $p_1(n)$ and $p_2(n)$, we define the fidelity as $\sum_n \sqrt{p_1(n)p_2(n)}$. The fidelity decreases as the mean photon number $\bar{n}$ increases, whereas increasing B extends the range of mean photon numbers that can be resolved with high fidelity. At the mean photon number $\bar{n} = 4.1$ corresponding to the input thermal field characterized in Figure 1**d**, the detector model retains a fidelity of approximately 97%. Fig. S2**b**-**d** compare the ideal and time-binned photon-number distributions for representative mean photon numbers and show that the two distributions become increasingly different as $\bar{n}$ approaches B. These results show that time binning of click-detector events provides an effective approximation to ideal photon-number-resolved detection for thermal states in the operating regime relevant to this work. The loss of fidelity at larger mean photon numbers can, in principle, constrain the scalability of the present implementation; however, this limitation can be mitigated by increasing the number of temporal bins, introducing detector multiplexing, or increasing the number of PNR detection channels.

6 | Note 6: Tradeoff Between Measurement Time and Photon Number Post-Selection

We next quantify the tradeoff between the number of measurements and the precision with which a selected photon-number probability can be estimated. We assume that the photon-number-resolving measurement contains a sufficient number of bins for its POVM expectation values to reproduce those of an ideal photon-number-resolving detector. Under this condition, a thermal state with mean photon number $\bar{n}$ has photon-number probability

$$p(\bar{n}, N) = \mathrm{Tr}[\hat{\rho}\hat{n}_N] = \frac{\bar{n}^N}{(1+\bar{n})^{N+1}}. \tag{S63}$$

Experimentally, this probability is estimated from M independent measurements by calculating the fraction of outcomes in which N photons are observed. In our implementation, one measurement requires 1 μs, so a dataset containing $M = 10^6$ measurements corresponds to an acquisition time of 1 s. For a fixed photon number N, each measurement can be represented by a binary random variable X_N^i that takes the value 1 with probability $p(\bar{n}, N)$ and 0 with probability $1 - p(\bar{n}, N)$.
We first characterize the statistics of one measurement. Its expectation value is

$$\langle X_N^i \rangle = 1 \cdot [p(\bar{n}, N)] + 0 \cdot [1 - p(\bar{n}, N)] = p(\bar{n}, N). \tag{S64}$$

The corresponding second moment is

$$\langle (X_N^i)^2 \rangle = 1^2 \cdot [p(\bar{n}, N)] + 0^2 \cdot [1 - p(\bar{n}, N)] = p(\bar{n}, N). \tag{S65}$$

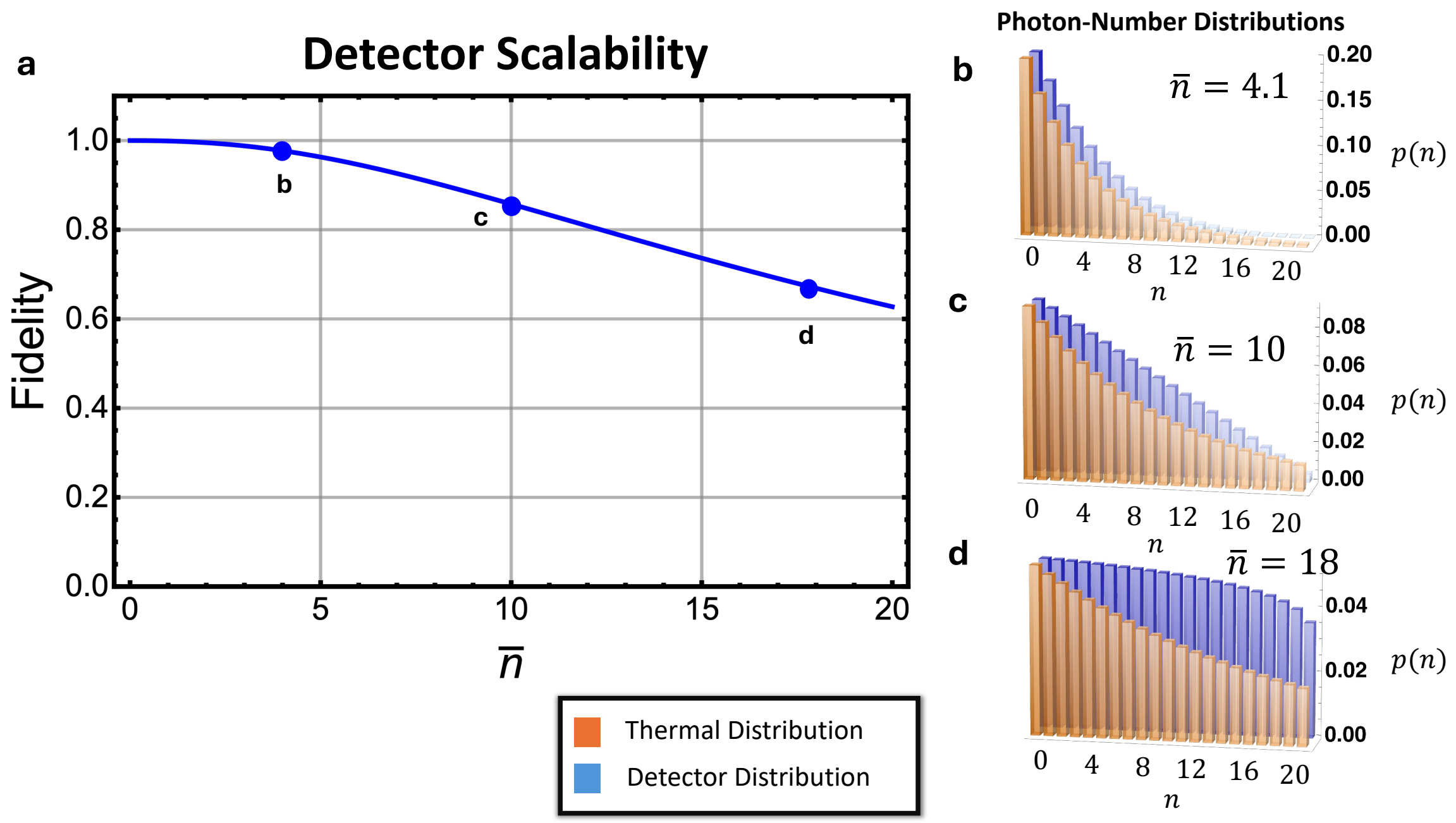

FIGURE S2 | **Fidelity and scalability of time-binned photon-number-resolving detection.** We evaluate the ability of the time-binned detection scheme to reproduce the photon-number statistics obtained with an ideal photon-number-resolving detector for thermal light. The calculation uses $B = 20$ temporal bins. Panel **a** shows the fidelity between the time-binned distribution and the ideal thermal photon-number distribution as a function of the mean photon number $\bar{n}$; the value $\bar{n} = 4.1$ corresponding to the input thermal field characterized in Figure 1**d** is marked explicitly. Panels **b**-**d** compare the ideal thermal distribution (orange) with the distribution reconstructed by the time-binned detector model (blue) for $\bar{n} = 4.1$, 10, and 18, respectively. In each panel, n denotes the post-selected photon number and $p(n)$ is the probability of observing that outcome.

The standard deviation of a single measurement is therefore

$$\begin{aligned}\sigma(X_N^i) &= \sqrt{\langle (X_N^i - \langle X_N^i \rangle)^2 \rangle} \\ &= \sqrt{\langle (X_N^i)^2 \rangle - \langle X_N^i \rangle^2} \\ &= \sqrt{p(\bar{n}, N) - p^2(\bar{n}, N)} \\ &= \sqrt{p(\bar{n}, N)(1 - p(\bar{n}, N))}.\end{aligned} \tag{S66}$$

We now form the estimator obtained by averaging the M measurement outcomes,

$$\hat{X}_N = \frac{1}{M} \sum_{i=1}^{M} X_N^i. \tag{S67}$$

The mean of this estimator is

$$\langle \hat{X}_N \rangle = \frac{1}{M} \sum_{i=1}^{M} \langle X_N^i \rangle = \frac{1}{M} \sum_{i=1}^{M} p(\bar{n}, N) = p(\bar{n}, N). \tag{S68}$$

As expected, averaging over M measurements does not change the mean. We next evaluate the second moment,

$$\begin{aligned}\langle \hat{X}_N^2 \rangle &= \frac{1}{M^2} \sum_{i,j=1}^{M} \langle X_N^i X_N^j \rangle \\ &= \frac{1}{M^2} \left(\sum_{i=1}^{M} \langle (X_N^i)^2 \rangle + \sum_{i \neq j} \langle X_N^i X_N^j \rangle \right) \\ &= \frac{1}{M^2} \left(\sum_{i=1}^{M} p(\bar{n}, N) + \sum_{i \neq j} p^2(\bar{n}, N) \right) \\ &= \frac{1}{M^2} \left(M p(\bar{n}, N) + (M^2 - M) p^2(\bar{n}, N) \right) \\ &= \frac{p(\bar{n}, N)(1 + (M-1) p(\bar{n}, N))}{M}.\end{aligned} \tag{S69}$$

The standard deviation of the full estimator is then

$$\begin{aligned}\sigma(\hat{X}_N) &= \sqrt{\langle \hat{X}_N^2 \rangle - \langle \hat{X}_N \rangle^2} \\ &= \sqrt{\frac{p(\bar{n}, N)(1 + (M-1) p(\bar{n}, N))}{M} - p^2(\bar{n}, N)} \\ &= \sqrt{\frac{p(\bar{n}, N)(1 - p(\bar{n}, N))}{M}}.\end{aligned} \tag{S70}$$

Thus, the statistical uncertainty of the estimate of $p(\bar{n}, N)$ scales as $1/\sqrt{M}$ for every photon number N. Solving for the number of measurements required to reach a target absolute uncertainty gives

$$M = \frac{p(\bar{n}, N)(1 - p(\bar{n}, N))}{\sigma^2}, \tag{S71}$$

where $\sigma \equiv \sigma(\hat{X}_N)$. Because $p(\bar{n}, N)$ becomes very small for large N, it is often more informative to work with the relative error $\epsilon = \sigma / p(\bar{n}, N)$. Solving for the required number of measurements in terms of ϵ yields

$$M = \frac{1 - p(\bar{n}, N)}{\epsilon^2 \cdot p(\bar{n}, N)} = \frac{1 - \frac{\bar{n}^N}{(1+\bar{n})^{N+1}}}{\epsilon^2 \cdot \frac{\bar{n}^N}{(1+\bar{n})^{N+1}}} = \frac{(1 + \bar{n})^{N+1} - \bar{n}^N}{\epsilon^2 \cdot \bar{n}^N}, \tag{S72}$$

which directly quantifies the measurement-time cost of resolving increasingly large photon-number components with a prescribed relative precision. Fig. S3 plots the required number of measurements on a base-10 logarithmic scale as a function of the detected photon number for $\bar{n} = 4.1$. Both increasing the selected photon number and demanding higher precision require larger datasets. For example, a 1 s dataset containing $M = 10^6$ measurements is sufficient to estimate the 10-photon component with a relative error of 1%, whereas the 20-photon component is estimated with a relative error of approximately 5%. This analysis provides the quantitative measurement-time framework for photon-number post-selection in our multiphoton quantum-information platform and makes explicit the tradeoff between the size of the selected multiparticle subsystem, statistical precision, and acquisition time.

7 | Note 7: Performance and benchmarking of the multiphoton quantum reservoir

In this note, we characterize the computational performance of the multiphoton quantum reservoir introduced in the main text (Figure 4). We first place our platform in the context of other experimental demonstrations of photonic quantum reservoir computing. We then isolate the contribution of multiphoton statistics by contrasting our reservoir with a classical counterpart read out from the same measured field.

7.1 | A. Comparison with existing photonic quantum reservoir platforms

Table S1 collects recent experimental realizations of photonic quantum reservoir computing and our platform. These platforms are built for different problems: the Gaussian-boson-sampling reservoir of Cimini *et al.* [S5] performs image and spoken-vowel classification, whereas the quantum-memristor device of Selimović *et al.* [S6] and the reconfigurable interferometer of Di Bartolo *et al.* [S7] address the forecasting of temporal sequences. Our quantum reservoir is used to reconstruct arbitrary nonlinear functions. Since the metrics are fixed by the task (classification accuracy, mean-squared error, and coefficient of determination), we contrast the approaches in terms of the platform, the quantum resource, and the conditions under which they are operated. Note that the reservoirs of Refs. [S5, S6, S7]

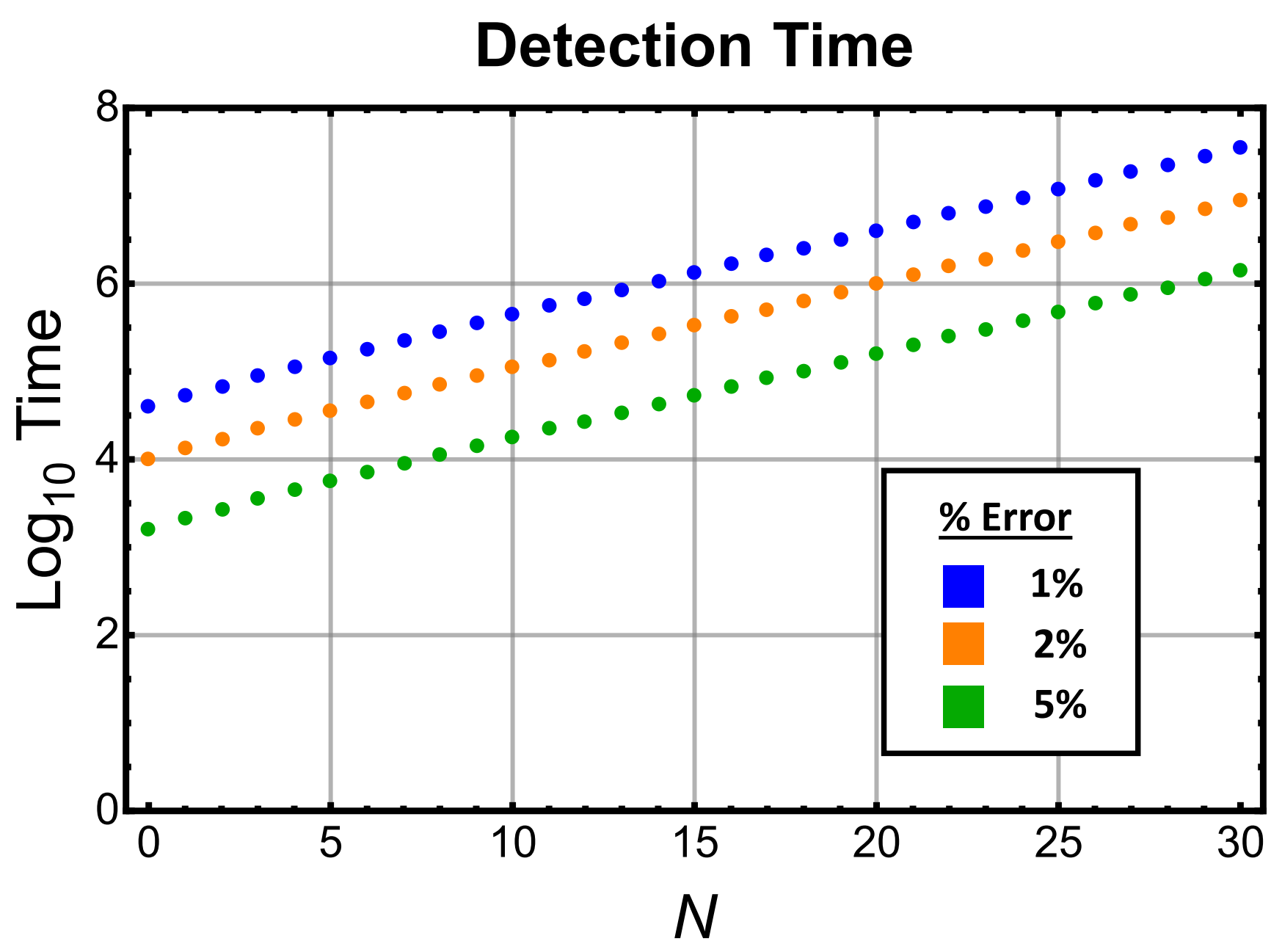

FIGURE S3 | **Measurement Time Tradeoff with Increasing Post-Selected Photon Number.** The required measurement time is plotted on a base-10 logarithmic scale as a function of the resolved photon number N for a thermal state with mean photon number $\bar{n} = 4.1$. Time is expressed in units of μs because each individual measurement requires 1 μs. Curves are shown for target relative errors of 1%, 2%, and 5%. Higher statistical precision requires larger datasets, while resolving larger photon-number components requires rapidly increasing measurement times.

TABLE S1 | Comparison of recent experimental photonic quantum reservoir computing platforms.

	**This work**	**Cimini *et al.***	**Selimović *et al.***	**Di Bartolo *et al.***
**Platform**	Free-space OAM + polarization multiphoton network	Frequency-multiplexed Gaussian boson sampler with >400 modes	Integrated photonic quantum memristor (Mach–Zehnder interferometer + feedback loop)	Reconfigurable integrated linear-optical circuit + active feedback loops
**Quantum resource**	Multiphoton Fock statistics distilled from classical thermal light	Squeezing + multimode correlations	Single photons + memristive nonlinearity	Two-photon indistinguishability (quantum correlations)
**Task type**	Reconstruction of arbitrary nonlinear functions	Classification	Temporal forecasting	Temporal forecasting
**Training time**	< 1 ms (specifically 30 μs)	Not reported	Not reported	Not reported
**Reported performance**	$R^2 = 0.94$–0.97 (reconstruction of six nonlinear functions)	Accuracy = 80%–100% (non-linearly separable multi-class)<br>Accuracy = 97% (vowels dataset; 30,000 frames)<br>Accuracy = 60% (MNIST dataset)	MSE = 2.8×10^{-5} (NARMA model)<br>MSE = 1.87×10^{-4} (Mackey–Glass model)<br>MSE = 1.26×10^{-2} (Santa Fe dataset)	Accuracy ≈ 1.0 (temporal XOR, delay $d = 1$–2)<br>Accuracy ≈ 0.7 (temporal XOR, $d = 3$)<br>Accuracy ≈ 0.5 (temporal XOR, $d = 4$)<br>MSE ≈ 2.7×10^{-3} to 5.9×10^{-3} (NARMA-N models)<br>MSE ≈ 6.49×10^{-3} and 4.94×10^{-3} (Mackey–Glass model)

rely on nonclassical inputs, squeezed light or heralded single photons, whose preparation and preservation place stringent demands. In contrast, our platform distills its multiphoton quantum states from classical thermal light by means of Fock projective measurements, inheriting the loss insensitivity of thermal fields. This allows the reservoir to operate at room temperature and remain functional despite the noise and losses.

7.2 | B. Classical and quantum reservoir

Additionally, we compare our quantum reservoir (861-dimensional vector provided by the Fock projective measurement) with a classical reservoir, which is obtained from the same optical field, retaining only the mean photon number in each of the 21 OAM modes.

TABLE S2 | Coefficient of determination R^2 for the reconstruction of the six nonlinear functions, obtained with the classical and quantum reservoirs. Values are averaged over random train/test partitions of the 91 input states.

**Target function**	**Classical reservoir**	**Quantum reservoir**
Quadratic	0.92	0.94
Cubic	0.89	0.93
Quartic	0.90	0.94
Exponential	0.96	0.97
Logarithmic	0.97	0.97
Damped sinusoid	0.69	0.94
**Mean**	**0.89**	**0.95**

This 21-dimensional vector is the information of the intensity measurement with no access to the photon-number distribution. In both cases, the mapping onto the target function is a single layer of linear regression, the only trained element of the model. As in the quantum case, 80% of the 91 input states is assigned to training, while the remaining 20% is used for testing. We report the coefficient of determination R^2 on the testing set, averaged over random partitions of the data. The results are shown in Table S2. The classical reservoir reaches an overall $R^2 = 0.89$, against $R^2 = 0.95$ for the quantum reservoir. Both the classical and quantum reservoirs accurately reconstruct monotonic targets. However, for strongly nonlinear functions (quartic and damped sinusoid functions) the coefficient of determination rises from 0.69 (classical reservoir) to 0.94 once the full photon distribution is retained (quantum reservoir). Because the two reservoirs are extracted from the same field, this difference is evidence of the robustness and computational value of the multiphoton quantum statistics distilled by our Fock projective measurement.

7.3 | C. Structure of the feature space

Since our reservoir maps each polarization-encoded input onto an 861-component vector, we study its effective dimension through a principal-component analysis of the 91×861 feature matrix produced by the Fock projective measurement. The feature matrix attains the maximal rank permitted by the 91 input states, so that every input is mapped onto a linearly independent state, that is, the map has full kernel rank, the standard measure of the separation property that a reservoir must possess in order to support a linear readout [S8]. Interestingly, the variance carried by these directions, however, is strongly concentrated: the leading principal component alone accounts for 75% of the total variance, and sixteen components are needed to reach 99%, so that the effective dimension given by the participation ratio of the eigenvalue spectrum is only 1.7. This low effective dimension is the ceiling imposed by the one-dimensional input rather than a collapse of the representation, in line with the general observation that reservoirs with high-dimensional state spaces exhibit effective dynamics bounded by the dimension of their input [S9]. What makes the map suitable for computing is therefore not a high effective dimension but its full kernel rank [S8], together with the curvature of this one-dimensional embedding. This structure also clarifies why the high nominal dimensionality does not harm generalization: with the signal confined to a low effective dimension and a ridge-regularized linear readout, the model does not overfit despite having more features (861) than training points, and the scores in Table S2 confirm that the reconstruction generalizes across all six functions.

7.4 | D. Robustness to noise

We identify two distinct sources of noise in our reservoir computing experiment. The first is the noise associated with the detection stage, namely the finite detection efficiency and the dark counts of our avalanche photodiodes, read out through a time tagger. Importantly, this source does not degrade the reservoir-computing performance as long as a sufficient number of samples is acquired. The photon-number distribution used to build the output vector is reconstructed statistically: the detection events are grouped into 1-μs windows, and 10^6 such windows are accumulated for each polarization-OAM setting during a 1-s acquisition (amounting to a raw acquisition time of about 32 min for the full 91×21 dataset). Because the linear readout is trained directly on these measured distributions, a stable detector response is absorbed into the fixed reservoir map, while the associated statistical fluctuations average down as the number of acquired windows increases. This behavior is consistent with the loss insensitivity of the multiphoton systems distilled from thermal light. The second source is the coupling between the OAM and photon-number degrees of freedom introduced by the multimode network. Because this coupling mixes many modes, it could at first sight be regarded as an uncontrolled source of disorder. In our scheme, however, it is the computational resource of the platform: it is deterministic and reproducible once the reservoir configuration is fixed and, crucially, it varies with the polarization of the input state. Since the input is encoded in polarization, this input-dependent coupling is precisely what maps each input onto a distinct, high-dimensional photon-number–OAM distribution, thereby generating the 861-component feature vector that the linear readout exploits.

References

[S1] Craig S Hamilton, Regina Kruse, Linda Sansoni, Sonja Barkhofen, Christine Silberhorn, and Igor Jex. Gaussian boson sampling. *Physical review letters*, 119(17):170501, 2017.

[S2] Riley B. Dawkins, Mingyuan Hong, Chenglong You, and Omar S. Magaña-Loaiza. The quantum gaussian–schell model: a link between classical and quantum optics. *Opt. Lett.*, 49(15):4242–4245, Aug 2024. doi: 10.1364/OL.520444.

[S3] Chenglong You, Mingyuan Hong, Fatemeh Mostafavi, Jannatul Ferdous, Roberto de J. León-Montiel, Riley B. Dawkins, and Omar S. Magaña-Loaiza. Isolating the classical and quantum coherence of a multiphoton system. *PhotoniX*, 5(1):39, 2024. ISSN 2662-1991. doi: 10.1186/s43074-024-00153-4.

[S4] Chenglong You, Mario A. Quiroz-Juárez, Aidan Lambert, Narayan Bhusal, Chao Dong, Armando Perez-Leija, Amir Javaid, Roberto de J. León-Montiel, and Omar S. Magaña-Loaiza. Identification of light sources using machine learning. *Appl. Phys. Rev.*, 7(2):021404, 2020. doi: 10.1063/1.5133846.

[S5] Valeria Cimini, Mandar M Sohoni, Federico Presutti, Benjamin K Malia, Shi-Yuan Ma, Ryotatsu Yanagimoto, Tianyu Wang, Tatsuhiro Onodera, Logan G Wright, and Peter L McMahon. Large-scale quantum reservoir computing using a gaussian boson sampler. *npj Quantum Information*, 12(104), 2026.

[S6] Mirela Selimović, Iris Agresti, Michał Siemaszko, Joshua Morris, Borivoje Dakić, Riccardo Albiero, Andrea Crespi, Francesco Ceccarelli, Roberto Osellame, Magdalena Stobińska, et al. Experimental neuromorphic computing based on quantum memristor. *arXiv preprint arXiv:2504.18694*, 2025.

[S7] Rosario Di Bartolo, Simone Piacentini, Francesco Ceccarelli, Giacomo Corrielli, Roberto Osellame, Valeria Cimini, and Fabio Sciarrino. Time-series forecasting with multiphoton quantum states and integrated photonics. *npj Quantum Information*, 12(91), 2026.

[S8] Robert Legenstein and Wolfgang Maass. Edge of chaos and prediction of computational performance for neural circuit models. *Neural networks*, 20(3):323–334, 2007.

[S9] Muhammed Fadera. On the dimension of pullback attractors in recurrent neural networks. *arXiv preprint arXiv:2501.11357*, 2025.
